# ROS-responsive H_2_S-releasing quaternized chitosan dental implant coating for improved soft tissue integration, antibacterial and immunomodulatory capacities

**DOI:** 10.1016/j.bioactmat.2026.07.056

**Published:** 2026-08-20

**Authors:** Yu Xie, Dandan Xia, Zhen Chen, Haitong Wu, Lepu Zeng, Ting Zhong, Wenxue Chen, Xinchen Du, Ming Ma, Xuechao Yang

**Affiliations:** a Department of Basic Oral Medicine, School and Hospital of Stomatology, Guangdong Engineering Research Center of Oral Restoration and Reconstruction & Guangzhou Key Laboratory of Basic and Applied Research of Oral Regenerative Medicine, Guangzhou Medical University, Guangzhou, 510182, China; b Department of Prosthodontics, Peking University School and Hospital of Stomatology, National Center for Stomatology, National Clinical Research Center for Oral Diseases, National Engineering Research Center of Oral Biomaterials and Digital Medical Devices, Beijing Key Laboratory for Intelligent Biomanufacturing and Regeneration of Craniofacial Tissues, Beijing, 100081, China; c Department of Chemical and Environmental Engineering, Hetao College, Bayannaoer, Inner Mongolia, 015000, China; d State Key Laboratory of Oral & Maxillofacial Reconstruction and Regeneration, National Clinical Research Center for Oral Diseases, Shaanxi Key Laboratory of Stomatology, Department of Prosthodontics, School of Stomatology, The Fourth Military Medical University, Xi'an, Shaanxi, 710032, China

**Keywords:** Dental implant, Soft tissue integration, Hydrogen sulfide, Anti-infection, Immunomodulation

## Abstract

Early and stable soft tissue integration is a prerequisite for the long-term success of dental implants. Crucially, the formation of this biological seal is closely associated with bacterial invasion and the modulation of the local immune microenvironment. Herein, we developed a reactive oxygen species (ROS)-responsive hydrogen sulfide (H_2_S)-releasing quaternized chitosan (QCS) coating on the transmucosal interface of Ti-6Al-4V implants, termed TC4/PQCS/H_2_S. This coating exhibited favorable biocompatibility, enhancing the adhesion of human gingival epithelial cells (HGECs) and human gingival fibroblasts (HGFs), and improving soft tissue compatibility. Additionally, the TC4/PQCS/H_2_S possessed potent antibacterial activity against *Escherichia coli*, methicillin-resistant *Staphylococcus aureus*, and oral anaerobic pathogens, with effective suppression of biofilm formation. Furthermore, the TC4/PQCS/H_2_S enhanced cellular antioxidant defense and activated the Nrf2 pathway. It also promoted macrophage polarization toward the pro-healing M2 phenotype, with the involvement of the PI3K-Akt signaling pathway. Overall, this study presents an efficient surface modification strategy that integrates cell-adhesive, antibacterial, antioxidant, and immunomodulatory functions, demonstrating great potential for soft tissue integration.

## Highlight

• ROS-responsive H_2_S-releasing coating was constructed on the transmucosal interface of Ti-6Al-4V implant.
• The coating exhibits favourable soft tissue integration, antibacterial and immunoregulatory performance.
• The coating enhances cellular antioxidant defense, activates the Nrf2 pathway, and promotes macrophage polarization toward the pro-healing M2 phenotype through the PI3K-Akt signaling pathway.

## Graphical abstract

**Figure:**
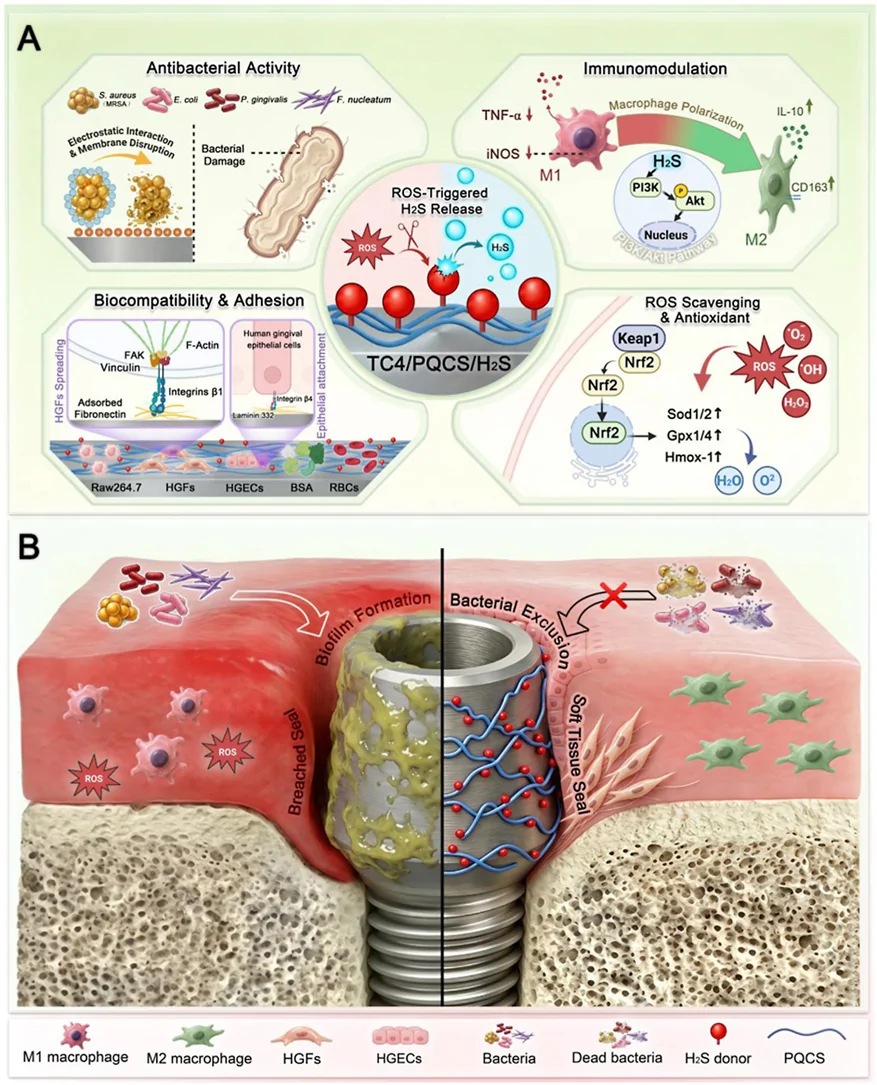

## 1 Introduction

Dental implant therapy is a widely utilized approach for rehabilitating missing teeth [1]. Despite significant advancements in osseointegration, research on soft tissue integration at the transmucosal interface remains relatively limited. Stable soft tissue integration acts as a defensive barrier that shields the underlying bone-implant interface, constituting an indispensable prerequisite for the long-term stability of implants [2]. Unlike natural teeth, the peri-implant mucosa lacks periodontal ligaments and cementum, featuring parallel fiber alignments that make the interface susceptible to bacterial invasion and inflammation [3]. Importantly, such pathological stimuli can trigger excessive reactive oxygen species (ROS) accumulation, which subsequently damages the extracellular matrix (ECM) and impairs the soft tissue seal [4]. Titanium alloys, particularly Ti-6Al-4V (TC4), are widely used in dental implants; however, they lack inherent antibacterial and immunomodulatory properties, leaving the transmucosal interface susceptible to microbial colonization and inflammatory damage [5]. To address these limitations, surface functionalization of TC4 implants to endow them with antibacterial, immunomodulatory, and pro-healing capacities represents a promising strategy for achieving stable soft tissue integration.

Immobilizing antimicrobial agents, such as copper (Cu^2+^), silver (Ag^+^), magnesium (Mg^2+^), zinc (Zn^2+^), or antibiotics, on implant surfaces has been proven to inhibit bacterial adhesion, proliferation, and biofilm formation [[6], [7], [8]]. However, uncontrolled ion release may induce cytotoxicity, and excessive antibiotic utilization tends to promote bacterial resistance [9,10]. Additionally, the accumulation of metal ions on the implant surface impairs human gingival fibroblasts (HGFs) adhesion and spreading by reducing cellular surface coverage [11]. To overcome the limitations of conventional metal ions and antibiotic agents, organic natural polymers with inherent antibacterial properties and superior cytocompatibility have emerged as a critical strategy. Quaternized chitosan (QCS), a cationic derivative of chitosan possessing permanent quaternary ammonium moieties, exhibits broad-spectrum antibacterial activity through a contact-killing mechanism that physically disrupts bacterial membranes [12]. This characteristic effectively prevents both the emergence of bacterial resistance and the local cytotoxicity commonly observed in metal-based antibacterial strategies. Moreover, chitosan-based materials exhibit favorable cytocompatibility, supporting the viability and proliferation of HGFs [13]. Despite these advantages, conventional QCS loading onto titanium substrates often relies on electrostatic attraction, which fails to provide sufficient interfacial bonding strength [14,15]. To address this issue, phloretic acid-modified quaternized chitosan (PQCS), fabricated via a tyrosinase-mediated biomimetic strategy, can strengthen interfacial bonding stability while maintaining antibacterial efficacy [16]. Nevertheless, the resulting implants still exhibit limited antioxidant and immunomodulatory capacities.

Excessive ROS in the peri-implant microenvironment exacerbates inflammatory responses and compromises the soft tissue seal [17]. Hydrogen sulfide (H_2_S) has emerged as a promising therapeutic agent due to its ability to effectively scavenge excessive ROS via the Keap1/Nrf2 pathway and to promote macrophage polarization toward the pro-healing M2 phenotype [[18], [19], [20]]. While H_2_S-modified implants have been developed, the physical adsorption of such donors onto TiO_2_ nanotube surfaces might suffer from weak interfacial stability and burst release risks [21]. To overcome these limitations, we propose the covalent immobilization of ROS-responsive H_2_S donors on the PQCS matrix via EDC/NHS-mediated amide condensation, which offers a promising strategy to achieve stable interfacial stability and preserve the functional integrity of the H_2_S-releasing system.

**Scheme 1:**
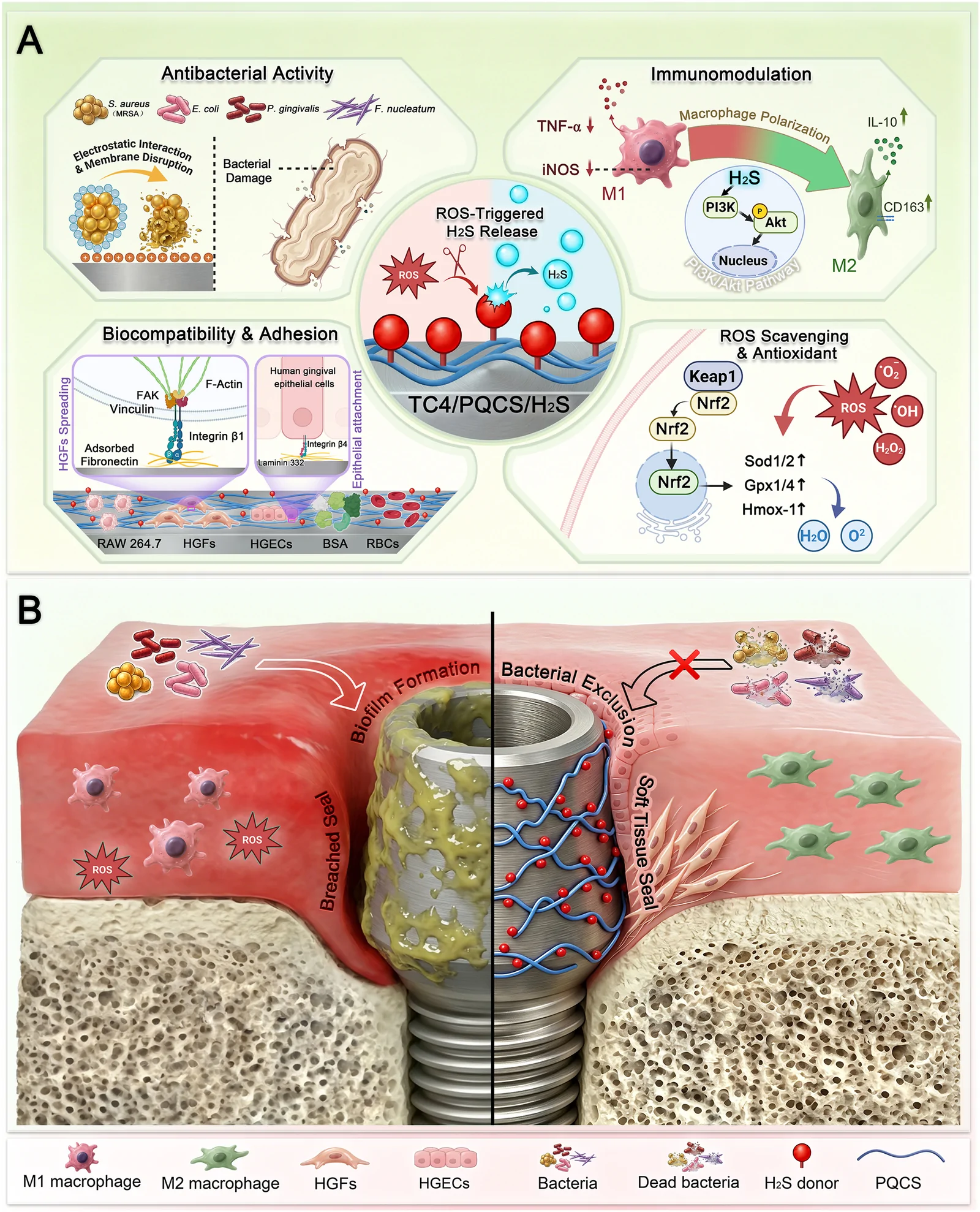
(A) The incorporation of PQCS endows the surface with prominent antibacterial properties and enhances the adhesion of human gingival fibroblasts (HGFs) and human gingival epithelial cells (HGECs). The integrated H_2_S donor further enhances the coating's antioxidant and immunomodulatory properties. (B) The unmodified implant neck is highly susceptible to bacterial invasion, compromised interfacial seal, and peri-implant mucositis, which may progress to irreversible tissue degradation. In contrast, the TC4/PQCS/H_2_S coating exhibits multifaceted biological functions by effectively supporting epithelial and fibroblast adhesion, displaying antibacterial activity, and promoting M2 macrophage polarization.

In this study, we developed a multifunctional surface modification strategy to endow the transmucosal interface of titanium implants with cell-adhesive, antibacterial, antioxidant, and immunomodulatory properties (Scheme 1). The fabrication involves two steps. First, inspired by the tyrosinase-mediated biomimetic coating method, PQCS was immobilized onto the TC4 surface to obtain the TC4/PQCS. Subsequently, an ROS-responsive H_2_S donor was covalently conjugated onto the TC4/PQCS via EDC/NHS-mediated amide condensation, yielding the TC4/PQCS/H_2_S. We hypothesized that the PQCS component could enhance antibacterial activity and cell adhesion, while the introduction of the H_2_S donor would further confer antioxidant and immunomodulatory capacities onto the modified surface. To verify these hypotheses, the cell adhesion ability, antibacterial activity, antioxidant capacity, and immunomodulatory effect of the TC4/PQCS/H_2_S were systematically evaluated *in vitro* and *in vivo*.

## 2 Results and discussion

### 2.1 Synthesis and characterization of PQCS and H_2_S donor

The phloretic acid-modified quaternized chitosan (PQCS) was synthesized using a two-step route (Fig. S1A). First, chitosan (CS) was modified with glycidyl trimethylammonium chloride (GTMAC) to synthesize quaternized chitosan (QCS), with a substitution degree of 31%. Second, the phloretic acid (PA) was covalently conjugated to QCS through carbodiimide-mediated amide coupling to yield PQCS. ^1^H NMR spectrum exhibited new chemical shifts at 7.2 (*a*) and 6.8 ppm (*b*), assignable to the methyne proton of the benzene ring in PA (Fig. S1B). Concurrently, chemical shifts at 4.6 (*c*) and 3.2 ppm (*d*) were observed, corresponding to the methine protons of the chitosan and the methyl protons of the quaternary ammonium group in GTMAC, respectively. These findings confirmed the successful synthesis of PQCS, which was further verified by ultraviolet visible (UV-vis) spectroscopy (Fig. S1C). Compared with the QCS solution, the PQCS solution exhibited a strong absorption peak at 278 nm, matching the characteristic absorbance of the PA solution.

The H_2_S donor was synthesized through a three-step route (Fig. S2A). First, p-benzylboronic acid pinacol ester bromide was reacted with thiourea to yield compound 1. Next, compound 1 underwent condensation with 2, 2′-dipyridyl disulfide to yield compound 2. Finally, compound 2 was reacted with 3-thiopropionic acid in a thiol-disulfide exchange to produce the H_2_S donor. The chemical structure was characterized by ^1^H NMR spectroscopy (Fig. S2B), showing characteristic chemical shifts at 7.74 (d, J = 8.0 Hz, 2H, -Ph-H) (*a*), 7.31 (d, J = 8.0 Hz, 2H, -Ph-H) (*b*), 3.86 (s, 2H, -Ph-CH_2_-S) (*c*), 2.66 (t, J = 6.6 Hz, 2H, -S-CH_2_-) (*d*), 2.59 (t, J = 6.6 Hz, 2H, -CH_2_-COOH) (*e*), and 1.32 (s, 12H, -C-(CH_3_)_2_) (*f*).

### 2.2 Fabrication and characterization of TC4/PQCS/H_2_S

**Fig. 1:**
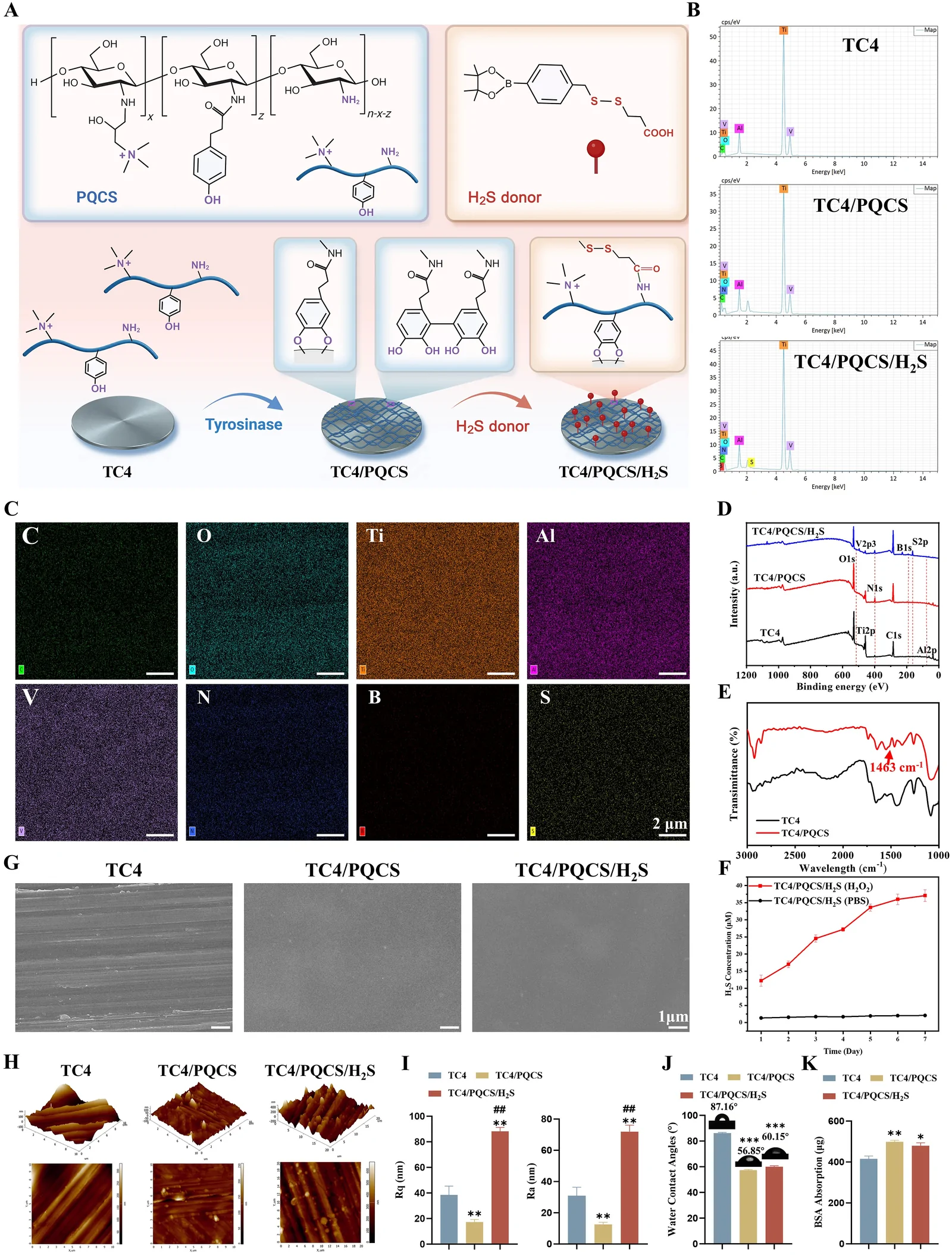
Fabrication, surface characterization, and H_2_S release behavior of TC4/PQCS/H_2_S. (A) PQCS was first adhered to the TC4 surface via tyrosinase-catalyzed reactions. The ROS-responsive H_2_S donor was then immobilized on the TC4/PQCS surface through EDC/NHS-mediated amide condensation, yielding the final TC4/PQCS/H_2_S. (B) EDS spectra of TC4, TC4/PQCS, and TC4/PQCS/H_2_S. (C) EDS elemental mapping image of TC4/PQCS/H_2_S. (D) XPS wide-scan spectra of the TC4, TC4/PQCS, and TC4/PQCS/H_2_S surfaces. (E) ATR-FTIR spectra. (F) The release of H_2_S from TC4/PQCS/H_2_S under different conditions. (G) SEM images of the samples. (H-I) AFM images of the samples and quantification of surface roughness parameters (Ra and Rq) for the samples. (J-K) Water contact angles and BSA adsorption capacities of the samples. Data are presented as mean ± SD (n = 3). *p < 0.05, **p < 0.01, and ***p < 0.001 versus TC4; **^##^***p* < 0.01 versus TC4/PQCS.

As shown in Fig. 1A, PQCS was immobilized onto the TC4 surface under the catalysis of tyrosinase to yield the TC4/PQCS. Energy-dispersive spectroscopy (EDS) and X-ray photoelectron spectroscopy (XPS) analyses confirmed the successful immobilization, as evidenced by an increased nitrogen (N) signal on the TC4/PQCS compared with that on pristine TC4 (Fig. 1B and D, Fig. S3A). Furthermore, the X-ray diffraction (XRD) patterns of the TC4/PQCS and TC4/PQCS/H_2_S groups revealed additional diffraction peaks characteristic of chitosan, verifying the presence of the PQCS coating on the TC4 surface (Fig. S3B). ATR-FTIR analysis further confirmed this structure by revealing a characteristic peak at 1463 cm^−1^, corresponding to the quaternary ammonium groups of PQCS (Fig. 1E). The modification mechanism of PQCS is primarily driven by tyrosinase-mediated catechol coating [16]. Specifically, tyrosinase rapidly oxidizes phenol groups to catechol and o-quinone moieties, endowing phenol-conjugated polymers with surface adhesion. This oxidation process also generates ROS (O^2−^, H_2_O_2_, •OH) and semiquinone, which catalyze both PQCS-PQCS and PQCS-TC4 cross-linking (Fig. 1A). Following PQCS modification, the H_2_S donor was covalently conjugated to the TC4/PQCS surface. EDS and XPS analyses revealed distinct boron (B) and sulfur (S) signals on the TC4/PQCS/H_2_S (Fig. 1B–D), which were absent in both the TC4 and TC4/PQCS substrates, thereby demonstrating the successful conjugation of the H_2_S donor. All aforementioned results confirmed the successful fabrication of the TC4/PQCS/H_2_S.

The controlled release behavior of H_2_S was evaluated employing an H_2_S kit. When exposed to H_2_O_2_, the TC4/PQCS/H_2_S demonstrated sustained H_2_S release, yielding approximately 37 μM of H_2_S after 7 days of incubation (Fig. 1F). These findings indicate that the TC4/PQCS/H_2_S coating enables controlled release of H_2_S in response to H_2_O_2_. The underlying mechanism is that upon exposure to H_2_O_2_ or ROS, the boronate ester protective groups on the donor are cleaved, forming an unstable intermediate. This intermediate subsequently collapses to generate a 1,4-quinone methide and a persulfide, which then slowly releases H_2_S [[22], [23], [24], [25], [26]].

The physical structure, topography, and roughness of an implant surface critically influence protein adsorption kinetics and cell adhesion [27,28]. The physical structure of the samples was characterized using a scanning electron microscope (SEM) (Fig. 1G). The untreated TC4 surface presented a morphology characterized by machining grooves or irregular ridges originating from the manufacturing and polishing processes. After PQCS modification, the surface became smoother, a phenomenon attributable to the pseudoplastic behavior of the chitosan solution and its superior film-forming property [29]. This viscous polymeric solution tends to fill the microscopic grooves inherently present on the pristine TC4 surface. To further elucidate the surface topography, atomic force microscopy (AFM) was employed, with the quantitative roughness parameters (Ra and Rq) summarized in Fig. 1H–I. The TC4/PQCS exhibited Ra and Rq values (12.65 ± 2.20 nm and 17.27 ± 3.34 nm, respectively) that were lower than those of the TC4 (30.95 ± 9.42 nm and 38.50 ± 11.92 nm). In contrast, the TC4/PQCS/H_2_S exhibited significantly higher Ra (71.96 ± 7.23 nm) and Rq (88.18 ± 5.34 nm) values, indicating a much rougher surface morphology.

The surface wettability plays a critical role in regulating both protein adsorption and subsequent cell adhesion [30]. The surface wettability of the sample was characterized using water contact angle measurements. Both TC4/PQCS and TC4/PQCS/H_2_S showed lower water contact angles than TC4 (Fig. 1J), indicating a marked enhancement in surface hydrophilicity. This enhancement originates from the successful incorporation of hydrophilic hydroxyl, amino, and quaternary ammonium groups onto the PQCS backbone [31]. Intriguingly, while AFM analysis revealed that conjugating H_2_S donor increased the nanoscale surface roughness, this topographical change did not translate into an enhanced surface wettability compared with TC4/PQCS. This phenomenon highlights the dominant role of surface chemistry over physical topography in this matrix. Mechanistically, conjugating the H_2_S donor onto PQCS consumes its hydrophilic amino groups while simultaneously exposing hydrophobic, methyl-rich pinacol and aromatic ring moieties. Consequently, the intrinsic hydrophobicity of these newly exposed chemical groups offset the expected wetting enhancement normally conferred by increased surface roughness.

Upon *in vivo* implantation, the substrate surface is rapidly coated by blood and interstitial fluid proteins, such as fibronectin, vitronectin, albumin, and fibrinogen, consequently forming a signaling layer/interface that directs cell behavior [32]. Both TC4/PQCS and TC4/PQCS/H_2_S showed higher bovine serum albumin (BSA) adsorption capacity than the TC4 (Fig. 1K), which is attributed to the integration of PQCS. As a cationic polymer enriched with quaternary ammonium groups, PQCS creates a positively charged surface, enhancing electrostatic attraction toward negatively charged proteins. Moreover, adhesive proteins such as fibronectin contain RGD sequences, which serve as specific ligands for cell attachment receptors [33]. Therefore, the PQCS coating may facilitate a favorable environment to support subsequent cell adhesion.

The interfacial stability of the coatings was evaluated using ultrasonic cleaning and 3M Scotch tape peeling tests. The SEM results showed that both the TC4/PQCS and TC4/PQCS/H_2_S coatings remained largely intact on the surface after high-frequency ultrasonication (50 kHz, 200 W), demonstrating great resistance to mechanical disturbance (Fig. S4A). Similarly, after three repeated peel cycles with a commercial 3M tape (adhesion strength > 1.23 N/cm), the coatings remained stably attached to the substrate without obvious peeling (Fig. S4B).

Considering the complicated oral environment *in vivo*, the long-term chemical stability of the functionalized substrates was further assessed after immersion in various physiologically relevant media for up to 14 days. Specifically, the samples were incubated in artificial saliva, PBS (pH 7.4), and acidic PBS (pH 5.5). As illustrated in Fig. S4C–D, SEM analysis indicated that both the TC4/PQCS and TC4/PQCS/H_2_S coatings maintained their structural integrity without cracking or peeling, with minor deposition of salt- and protein-like particulates derived from the immersion media observed on the surface. XPS analysis at 14 days further verified the surface chemical composition, with consistent N, B, and S signals detected (Fig. S5A). In addition, XRD patterns still exhibited the characteristic diffraction peaks of chitosan after 14 days of immersion, along with after ultrasonication and 3M tape peeling, suggesting that the crystalline structure of the coatings remained stable (Fig. S5B–F). Concurrently, water contact angle measurements were performed on samples under the same conditions, and the results demonstrated that the enhanced surface wettability of the modified groups remained stable, with negligible variations compared to their initial states (Fig. S5G–N). Notably, after 7 and 14 days of immersion in artificial saliva containing mucin, the water contact angles of TC4/PQCS and TC4/PQCS/H_2_S decreased significantly, indicating an increase in surface hydrophilicity. This change may be attributed to the adsorption of mucin molecules that expose polar groups (e.g., –OH, –COOH, and –NH_2_), which reduce the solid–air–liquid interfacial tension [34,35].

This great stability and durability of the coatings can be attributed to the specific chemical bonding within the system. Distinct from conventional chitosan coatings that rely on weak physical adsorption, PQCS forms strong covalent bonds with the TC4 substrate under tyrosinase catalysis, providing a stable interfacial anchor [16]. Moreover, the stable chemical conjugation between the H_2_S donor and the PQCS matrix significantly enhances the structural and functional stability of the coating. Collectively, these findings demonstrate that the TC4/PQCS and TC4/PQCS/H_2_S coatings possess potential resistance to mechanical abrasion and exceptional structural integrity during long-term immersion, suggesting their promise for further evaluation in clinical applications.

### 2.3 *In vitro* and *in vivo* biocompatibility evaluation

Validating the cytocompatibility of modified surfaces represents a fundamental prerequisite for assessing their translational potential. A non-toxic interface ensures the survival and functional preservation of human gingival epithelial cells (HGECs) and human gingival fibroblasts (HGFs) during the critical early healing period, providing the cellular basis for subsequent soft tissue integration [6]. Similarly, the biocompatibility towards macrophages (RAW264.7) ensures their survival during the inflammatory phase, allowing these immune cells to modulate the microenvironment and protect the formed seal from inflammatory breakdown [2].

**Fig. 2:**
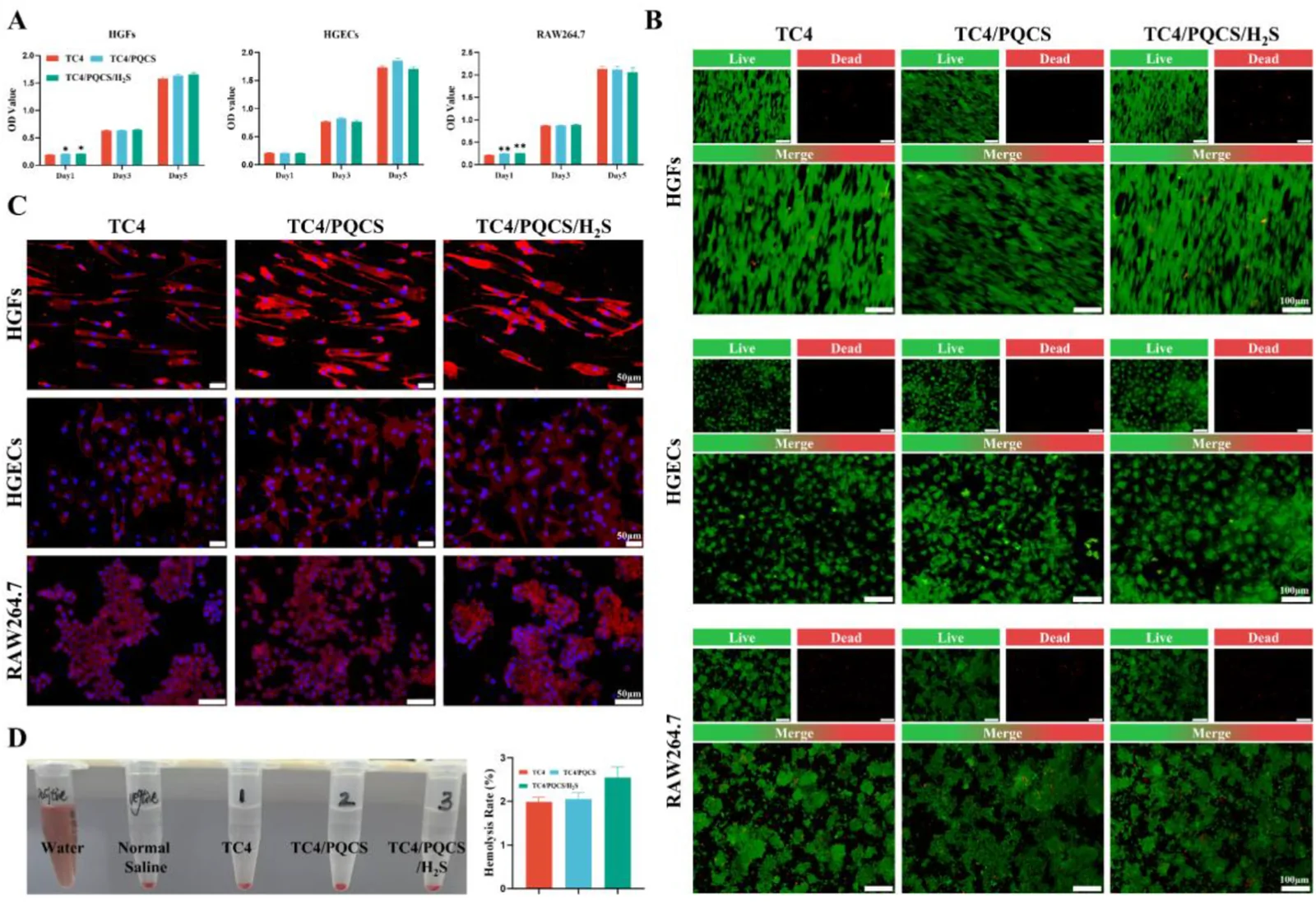
*In vitro* biocompatibility evaluation of the TC4, TC4/PQCS, and TC4/PQCS/H_2_S. (A) CCK-8 assay results of HGFs, HGECs, and RAW264.7 cells co-cultured on different samples for 1, 3, and 5 days. (B) Live/dead staining images of HGFs, HGECs, and RAW264.7 cells co-cultured on different samples for 3 days. (C) Fluorescence images of cytoskeleton staining for HGFs, HGECs, and RAW264.7 cells co-cultured on different samples for 24 h. (D) Photographs of the supernatants and the corresponding hemolysis rates. Data are presented as mean ± SD (n = 3). *p < 0.05 and **p < 0.01 versus TC4.

The proliferation kinetics of HGECs, HGFs, and RAW264.7 were evaluated using the CCK-8 assay at various time points. The OD values for all three cell types across all groups exhibited a continuous upward trend from day 1 to day 5 (Fig. 2A), indicating favorable biocompatibility. On day 1, the OD values for both HGFs and RAW264.7 macrophages in the TC4/PQCS and TC4/PQCS/H_2_S groups were significantly higher than those of the TC4 group (Fig. 2A). Live/dead staining further corroborated these findings (Fig. 2B). Fluorescence images confirmed that HGECs, HGFs, and RAW264.7 maintained high viability across all groups after a 3-day incubation period, verifying that the introduction of PQCS and H_2_S donor do not cause cytotoxicity under physiological conditions. Furthermore, cytoskeletal staining was performed to visualize cell adhesion morphology (Fig. 2C). HGFs cultured on TC4/PQCS and TC4/PQCS/H_2_S showed better proliferation than those on unmodified TC4. Similarly, HGECs cultured on TC4/PQCS and TC4/PQCS/H_2_S exhibited enhanced actin organization and spreading compared to TC4. For RAW264.7, cells exposed to the TC4/PQCS and TC4/PQCS/H_2_S exhibited adhesion morphology comparable to the TC4 group, demonstrating that the modified substrates sustain appropriate biocompatibility.

Hemocompatibility is another critical parameter for evaluating the biocompatibility of implants, as blood constitutes the primary physiological fluid encountered by the substrate upon clinical implantation [36]. Hemolysis rates were quantified in accordance with the ISO 10993-4 standard. The TC4, TC4/PQCS, and TC4/PQCS/H_2_S exhibited comparable hemolysis rates within an acceptable range (<5%) (Fig. 2D), substantiating favorable hemocompatibility. Additionally, hematoxylin and eosin (H&E) staining of major organs (including the heart, liver, spleen, kidneys, and lungs) revealed no observable structural abnormalities or systemic signs of toxicity (Fig. S6A), further supporting the favorable biosafety profile of the modified implants.

### 2.4 *In vitro* and *in vivo* cell adhesion assessment

Winning the “race for the surface” between host cells and bacteria is critical for preventing early peri-implant infections [37]. HGECs play a critical role by forming an epithelial barrier that acts as the first line of defense against microbial invasion [2]. As the principal ECM-producing cells in peri-implant connective tissue, HGFs deposit collagen fibrils and differentiate into myofibroblasts, thereby reinforcing the mechanical stability of the mucosal seal and preventing epithelial downgrowth [38]. Therefore, enhancing the adhesion and spreading of both HGECs and HGFs on implant surfaces is fundamental for achieving rapid soft tissue integration and establishing a durable biological seal.

**Fig. 3:**
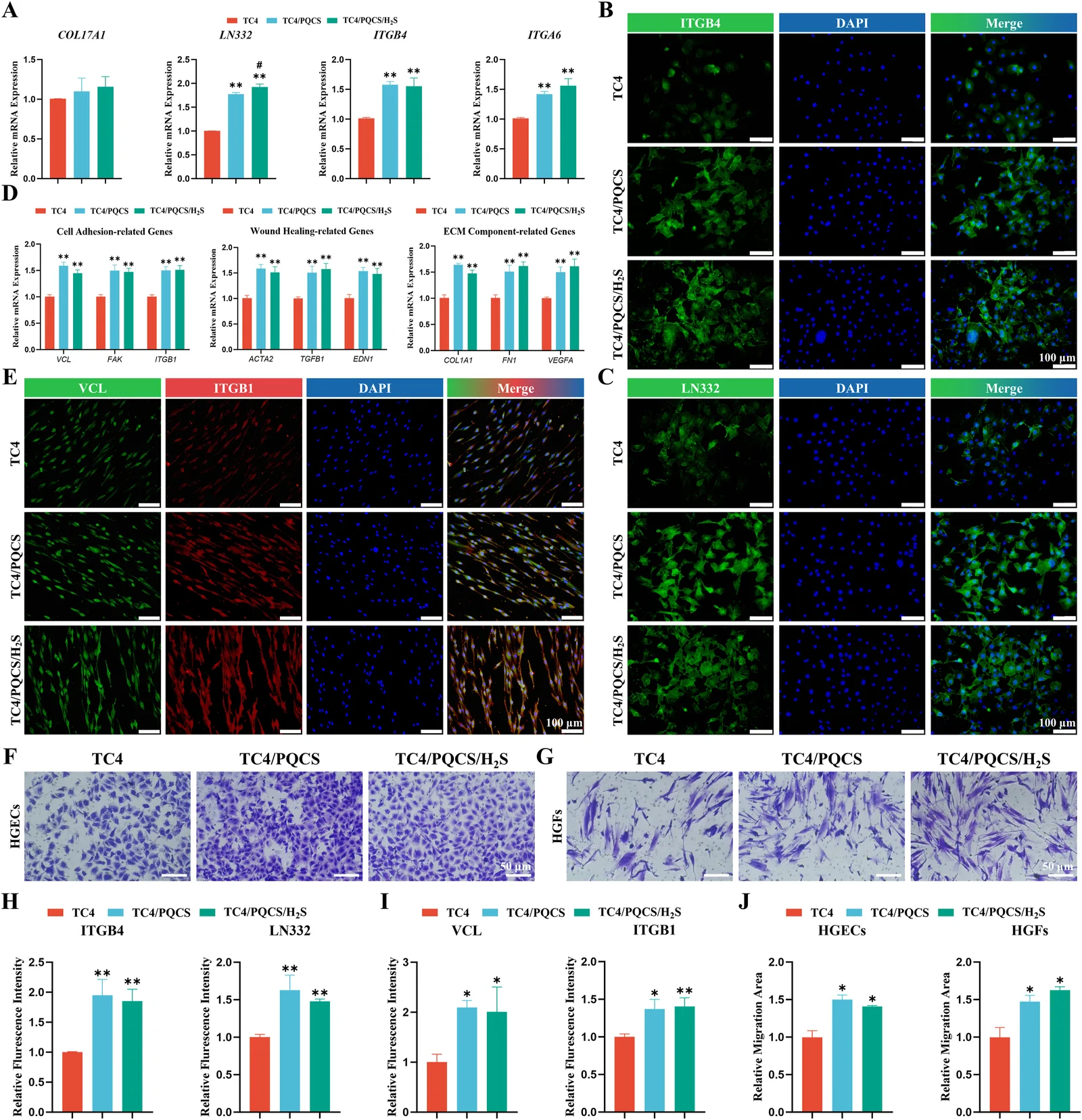
Impact of the samples on the adhesion behavior of HGECs and HGFs. (A) Relative mRNA expression levels of HGECs adhesion and hemidesmosome formation (*COL17A1*, *LN332*, *ITGB4*, and *ITGA6*) in HGECs co-cultured on the different samples for 24 h. (B-C) Fluorescence images of HGECs co-cultured on the different samples for 24 h. (D) Relative mRNA expression levels of HGFs adhesion-related genes (*VCL, FAK*, and *ITGB1*), wound healing-related genes (*ACTA2, TGFB1*, and *EDN1*), and ECM component-related genes (*COL1A1, FN1*, and *VEGFA*) in HGFs co-cultured on the different samples for 24 h. (E) Fluorescence images of HGFs co-cultured on the different samples for 24 h. (F, G) Representative crystal violet staining images showing the migration of HGECs and HGFs on different samples. (H) Quantitative analysis of relative fluorescence intensities for ITGB4 and LN332 in HGECs. (I) Quantitative analysis of relative fluorescence intensities for VCL and ITGB1 in HGFs. (J) Quantitative analysis of the relative migration areas for HGECs and HGFs. Data are presented as mean ± SD (n = 3). *p < 0.05 and **p < 0.01 versus TC4; **^#^***p* < 0.05 versus TC4/PQCS.

To assess the capacity of the modified surfaces to support an early epithelial barrier, the expression of genes regulating HGECs adhesion and hemidesmosome formation was evaluated. RT-qPCR analysis revealed that, although *COL17A1* expression showed no significant difference among the three groups, the expression levels of *LN332*, *ITGB4*, and *ITGA6* were significantly upregulated in the TC4/PQCS and TC4/PQCS/H_2_S groups relative to the TC4 group (Fig. 3A). Immunofluorescence staining further corroborated these findings, with higher protein levels of LN332 and ITGB4 detected in the two modified groups than in the TC4 group (Fig. 3B–C, H). LN332, a key basement membrane component, serves as the primary ligand for epithelial cell anchorage by engaging integrin receptors. ITGB4, as its cognate receptor, initiates the assembly of hemidesmosomal complexes that establish a stable mechanical connection between the epithelium and the underlying implant surface [39]. The coordinated upregulation of LN332 and ITGB4 suggests that the PQCS-modified surfaces provide a supportive microenvironment for epithelial adhesion and the formation of specialized anchoring structures.

The expression of genes related to adhesion (*VCL*, *FAK*, and *ITGB1*), wound healing (*ACTA2, TGFB1*, and *EDN1*), and ECM synthesis (*COL1A1, FN1,*and *VEGFA*) was quantified to investigate PQCS-mediated effects on soft tissue integration. All target genes in the TC4/PQCS and TC4/PQCS/H_2_S groups were upregulated relative to the TC4 group (Fig. 3D). This coordinated upregulation of adhesion- and ECM-related transcript levels points toward the potential capacity of the coatings in establishing soft tissue integration. Immunofluorescence analysis further supported these results (Fig. 3E, I), with the protein levels of both ITGB1 and VCL significantly elevated in the two modified groups compared with the TC4 group. To evaluate whether the coatings retained their pro-adhesive properties after 14 days of immersion in artificial saliva or PBS (pH 7.4 or 5.5), ITGB1 expression in HGFs cultured on the pretreated coatings was examined. The PQCS-coated groups consistently maintained higher ITGB1 expression than the TC4 group (Fig. S7A–D), suggesting that the coatings preserve their pro-adhesive capacity after clinically relevant solution pretreatment. ITGB1 acts as the primary transmembrane receptor, anchoring the cell to the modified surface and initiating intracellular adhesion signaling [40], whereas VCL is subsequently recruited to these sites to stabilize and strengthen focal adhesions by coupling the integrin receptors to the internal actin cytoskeleton [41]. Transwell migration assays further demonstrated that the TC4/PQCS and TC4/PQCS/H_2_S groups significantly enhanced the migratory capability of both HGECs and HGFs compared with the TC4 group (Fig. 3F–G, J), indicating that the PQCS component contributes to the enhanced cell migration.

**Fig. 4:**
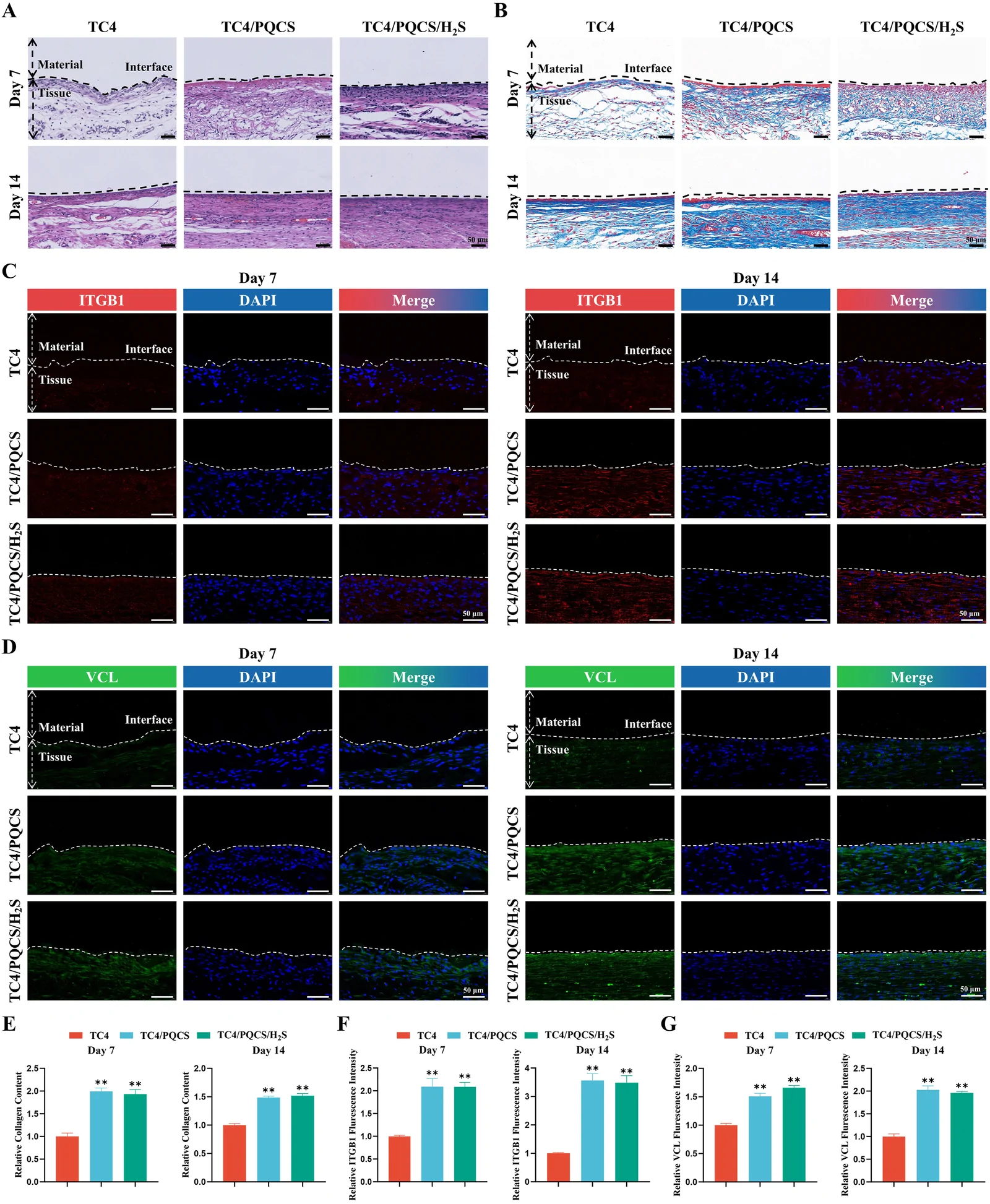
*In vivo* soft tissue response at the material-tissue interface. (A) H&E staining images of the material-tissue interface. (B) Masson staining images of relative collagen content. (C) Immunofluorescence staining of ITGB1 at the material-tissue interface at day 7 and day 14. (D) Immunofluorescence staining of VCL at the material-tissue interface at day 7 and day 14. (E) Quantitative analysis of the relative collagen content in the tissues adjacent to the implants. (F, G) Quantitative analysis of the relative fluorescence intensities for ITGB1 and VCL at the material-tissue interface. Data are presented as mean ± SD (n = 5). **p < 0.01 versus TC4.

The soft tissue response to the implants was further evaluated using a rat subcutaneous implantation model at 7 and 14 days post-implantation. H&E staining revealed favorable tissue responses across all groups, with no obvious pathological alterations observed at either time point (Fig. 4A). The TC4/PQCS and TC4/PQCS/H_2_S groups exhibited more organized tissue architecture adjacent to the implant interface compared with that in the TC4 group. Masson staining showed that the TC4 group presented loose and disrupted collagen fibers around the implant at both day 7 and day 14. In contrast, dense and continuous collagen deposition was observed around the TC4/PQCS and TC4/PQCS/H_2_S implants, with more evident organization and greater collagen density at day 14 compared to day 7 (Fig. 4B, E). Immunofluorescence staining of VCL and ITGB1 revealed significantly enhanced fluorescence intensities at the material–tissue interface in both TC4/PQCS and TC4/PQCS/H_2_S groups relative to the TC4 group (Fig. 4C–D, F-G). Immunohistochemical staining of COL1A1 showed a consistent increasing trend (Fig. S8A and B), further demonstrating a supportive microenvironment for fibroblast activity and ECM deposition on the modified surfaces.

The enhanced biological performance of the PQCS-modified surfaces, including improved cell adhesion, migration, and tissue integration, is primarily attributed to the physicochemical properties of the PQCS matrix. The abundant hydrophilic groups in PQCS, such as hydroxyl, amino, and quaternary ammonium groups, enhance surface wettability and initial protein adsorption [31]. Concurrently, the polycationic nature of PQCS facilitates electrostatic interactions with negatively charged cell membranes [14], which subsequently support integrin-mediated cell attachment and proliferation.

### 2.5 *In vitro* antibacterial and antibiofilm performance

The implant neck serves as a transmucosal component linking the osseointegrated fixture to the oral cavity, which is inevitably exposed to a polymicrobial environment upon installation. Bacteria colonizing the implant before the establishment of a soft-tissue seal frequently form biofilms, triggering peri-implant mucositis and potentially culminating in severe peri-implant infections [42]. Once established, biofilms exhibit enhanced resistance to antibiotics and host immune clearance, making them difficult to eradicate and posing a persistent threat to peri-implant soft tissue integrity [43]. Therefore, conferring intrinsic antibacterial and antibiofilm activities on the implant is critical for inhibiting bacterial adhesion and enhancing long-term implant stability.

**Fig. 5:**
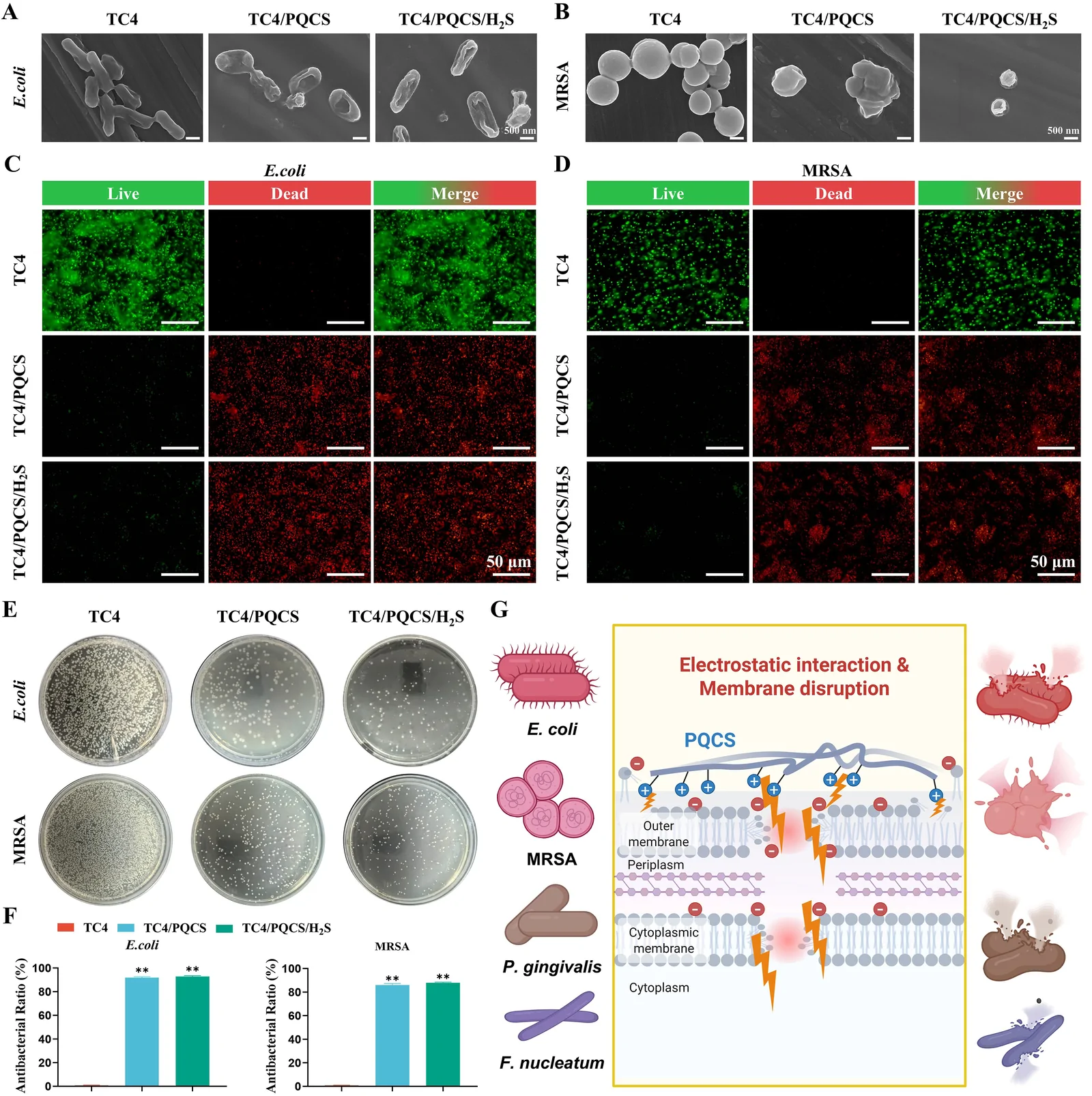
*In vitro* antimicrobial activity of the TC4, TC4/PQCS, and TC4/PQCS/H_2_S. (A-B) SEM and (C-D) live/dead staining images showed the morphology and viability of *E. coli* and MRSA after 24 h co-cultured with different samples. (E-F) Photographs of bacterial colonies and corresponding antibacterial rates for both bacteria in each group after 24 h incubation. (G) Graphic illustration of the *in vitro* antimicrobial activity, which was created with BioRender.com. Data are presented as mean ± SD (n = 3). **p < 0.01 versus TC4.

Antibacterial activity against *Escherichia coli* (*E. coli*) and methicillin-resistant *Staphylococcus aureus* (MRSA) was assessed via SEM observation, live/dead staining, and plate counting. Bacteria treated with the TC4/PQCS and TC4/PQCS/H_2_S exhibited severe structural destruction characterized by membrane wrinkling, perforation, and cytoplasmic leakage, whereas those exposed to the TC4 maintained a plump, intact morphology without obvious membrane disruption (Fig. 5A and B). Both TC4/PQCS and TC4/PQCS/H_2_S groups exhibited significantly higher numbers of dead bacteria (indicated by red fluorescence) and markedly reduced colony-forming units (CFU) (**p < 0.01) compared with the TC4 group (Fig. 5C–F). Following 14 days of immersion in artificial saliva and PBS (pH 7.4 and 5.5), as well as after ultrasonication and 3M tape peeling, the functionalized samples still exhibited reduced bacterial viability relative to the TC4 group (Fig. S9A–J). This indicates that the PQCS-modified coatings retain their antibacterial efficacy after mechanical challenges and exposure to clinically relevant solutions, suggesting their promise for further evaluation in oral environments. Given the clinical prevalence of common oral anaerobic bacteria, particularly *Porphyromonas gingivalis* (*P. gingivalis*) and *Fusobacterium nucleatum* (*F. nucleatum*), we further evaluated the antibacterial activity of the samples against these periodontal pathogens. Both TC4/PQCS and TC4/PQCS/H_2_S exhibited potent antimicrobial properties against these anaerobic strains (Fig. S10A and B).

The antibiofilm efficacy of TC4, TC4/PQCS, and TC4/PQCS/H_2_S against MRSA, *E. coli*, *P. gingivalis*, and *F. nucleatum* biofilms established on material surfaces was evaluated using crystal violet staining, with the biomass quantified by OD_590_ measurement (Fig. S10C and D). The TC4 group exhibited substantial biofilm formation across all four bacterial strains, as reflected by high crystal violet retention. In contrast, both PQCS-modified groups exhibited notable decreases in biofilm biomass. Specifically, against MRSA, the TC4/PQCS group achieved a biomass reduction of approximately 74.8%, while the TC4/PQCS/H_2_S group showed a comparable decline of 75.9%. For *E. coli*, the biomass was reduced by 74.9% for TC4/PQCS and by 75.4% for TC4/PQCS/H_2_S. When incubated with *P. gingivalis*, the TC4/PQCS surface decreased biofilm formation by 76.1%, and the TC4/PQCS/H_2_S surface exhibited a suppression of 74.7%. For *F. nucleatum*, the corresponding values were 75.0% for TC4/PQCS and 72.3% for TC4/PQCS/H_2_S. These findings collectively demonstrate that TC4/PQCS and TC4/PQCS/H_2_S display similar antibiofilm performance against all tested strains.

These results demonstrate that both TC4/PQCS and TC4/PQCS/H_2_S exhibit superior antibacterial and antibiofilm activities, which are primarily driven by the contact-killing mechanism of the PQCS matrix (Fig. 5G). PQCS contains positively charged quaternary ammonium groups that interact electrostatically with the negatively charged bacterial membrane, disrupting their structural integrity and triggering rapid leakage of intracellular components [12]. Although H_2_S possesses potential antibacterial activity, the cumulative release concentration within the 24-h evaluation period reached only approximately 10 μM (Fig. 1F), a level below the effective threshold required to exert significant antibacterial effects. Therefore, the contact-killing effect of PQCS predominated in the initial stage. Crucially, PQCS not only promotes preferential adhesion of HGFs and HGECs but also effectively inhibits bacterial adhesion and biofilm formation. This enables the pro-adhesive property of the coating to be fully realized, thereby contributing to early soft tissue seal formation.

### 2.6 Antioxidant activity and responsive-H_2_S release

Establishing strong initial adhesion and inhibiting bacterial invasion are prerequisites for effective soft tissue sealing. However, excessive ROS, triggered by acute inflammation and bacterial invasion after implantation, could impair postoperative healing [44]. ROS induces direct oxidative damage to HGECs and HGFs, disrupting cytoskeletal organization and impairing focal adhesion stability [17,45]. Moreover, ROS acts as a pro-inflammatory signal in macrophages, promoting ECM degradation [46]. An ideal implant surface should therefore possess active antioxidant activity to maintain a pro-healing microenvironment.

**Fig. 6:**
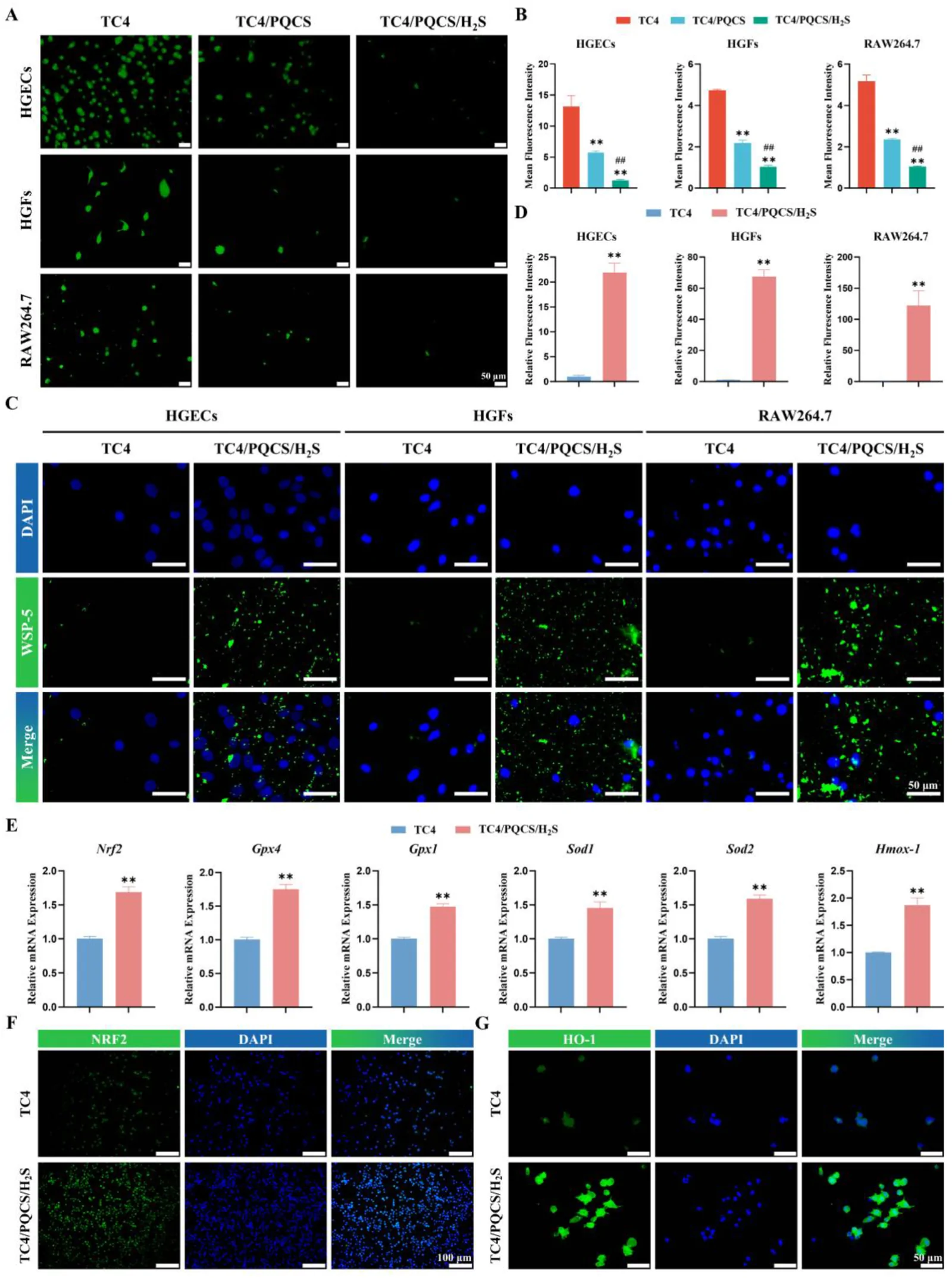
Antioxidative property of the TC4/PQCS/H_2_S. (A) Representative immunofluorescence images showing intracellular ROS levels in HGECs, HGFs, and RAW264.7 cells after incubation on different samples and treatment with H_2_O_2_. (B) Quantitative analysis of the mean fluorescence intensity of intracellular ROS in the three cell types. (C) Immunofluorescence images showing intracellular H_2_S generation tracked by the WSP-5 probe in HGECs, HGFs, and RAW264.7 cells. (D) Quantitative analysis of the relative fluorescence intensity of intracellular H_2_S in the three cell types. (E) Relative mRNA expression levels of antioxidant-related genes (*Nrf2*, *Gpx4*, *Gpx1*, *Sod1*, *Sod2*, and *Hmox-1*) in RAW264.7 cells cultured on TC4 and TC4/PQCS/H_2_S samples. (F, G) Representative immunofluorescence images of NRF2 nuclear translocation and HO-1 expression in RAW264.7 cells. Data are presented as mean ± SD (n = 3). **p < 0.01 versus TC4; ^##^*p* < 0.01 versus TC4/PQCS.

Under H_2_O_2_-induced oxidative stress, pronounced intracellular ROS accumulation was observed in the TC4 group. The TC4/PQCS group exhibited a slight decrease in ROS fluorescence, and the lowest fluorescence intensity was detected in the TC4/PQCS/H_2_S group (Fig. 6A and B). These results demonstrated that the incorporation of PQCS and ROS-responsive H_2_S release synergistically enhanced the antioxidant performance of the coating. The contribution of H_2_S to this effect was verified using the WSP-5 probe, with increased H_2_S fluorescence confirming the ROS-responsive release mechanism (Fig. 6C and D). To investigate the underlying pathways associated with H_2_S-mediated macrophage modulation, the Nrf2 signaling pathway was examined. The mRNA expression of *Nrf2* was upregulated in the TC4/PQCS/H_2_S group, which was accompanied by the marked upregulation of downstream antioxidant enzymes, including *Sod1*, *Sod2*, *Gpx1*, *Gpx4*, and *Hmox-1* (Fig. 6E). While *Keap1* mRNA levels showed no significant change, immunofluorescence staining revealed lower KEAP1 protein intensity in the TC4/PQCS/H_2_S group compared with the TC4 group (Fig. S11A–C), suggesting increased KEAP1 degradation and a subsequent reduction in its inhibitory effect on downstream signaling. In addition, the TC4 groups showed relatively weak NRF2 fluorescence with a diffuse cytoplasmic distribution. In contrast, the TC4/PQCS/H_2_S group showed increased NRF2 fluorescence intensity with nuclear translocation (Fig. 6F, Fig. S11D). Higher HO-1 intensity in the TC4/PQCS/H_2_S group suggested an effective downstream functional response (Fig. 6G, Fig. S11E). To further assess the functional involvement of this Nrf2 activation in modulating the antioxidant effect, macrophages were pretreated with the Nrf2-specific inhibitor ML385. As shown in Fig. S11F–G, ML385 treatment partially attenuated the ROS-scavenging effect of the TC4/PQCS/H_2_S coating, as evidenced by increased ROS fluorescence intensity.

Collectively, these findings suggest that the TC4/PQCS/H_2_S coating modulates the Nrf2 pathway to initiate the antioxidant response. This activation is partially attributed to H_2_S release, which exhibits intrinsic antioxidant activity by scavenging ROS and enhancing the cellular antioxidant defense system [20]. Nrf2 represents a core transcription factor closely linked to the cellular oxidative stress response. Following dissociation from Keap1, Nrf2 translocates into the cell nucleus and binds to the antioxidant response element (ARE) to initiate a series of downstream transcriptions, thereby synergistically upregulating the cellular antioxidant defense capacity [47]. Therefore, the upregulation and nuclear translocation of Nrf2 indicates an enhancement of antioxidant activity and the recovered redox homeostasis of RAW264.7, ensuring the viability and normal physiological functions of peri-implant immune cells.

### 2.7 The gradient anti-inflammatory effect of TC4/PQCS/H_2_S

Implant-soft tissue stability is critical for preventing bacterial invasion and subsequent tissue destruction. Macrophages, as key participants in innate immunity, play a dual role in this process [21]. The pro-inflammatory M1 phenotype (characterized by the expression of iNOS, CD86, IL-6, and TNF-α) is responsible for exacerbating tissue damage, whereas the anti-inflammatory M2 phenotype (characterized by the expression of ARG-1, IL-10, CD163, and TGF-β) drives tissue healing and angiogenesis [48]. An ideal transmucosal implant coating should therefore promote macrophage polarization toward pro-healing M2 phenotype to establish a favorable immune microenvironment.

**Fig. 7:**
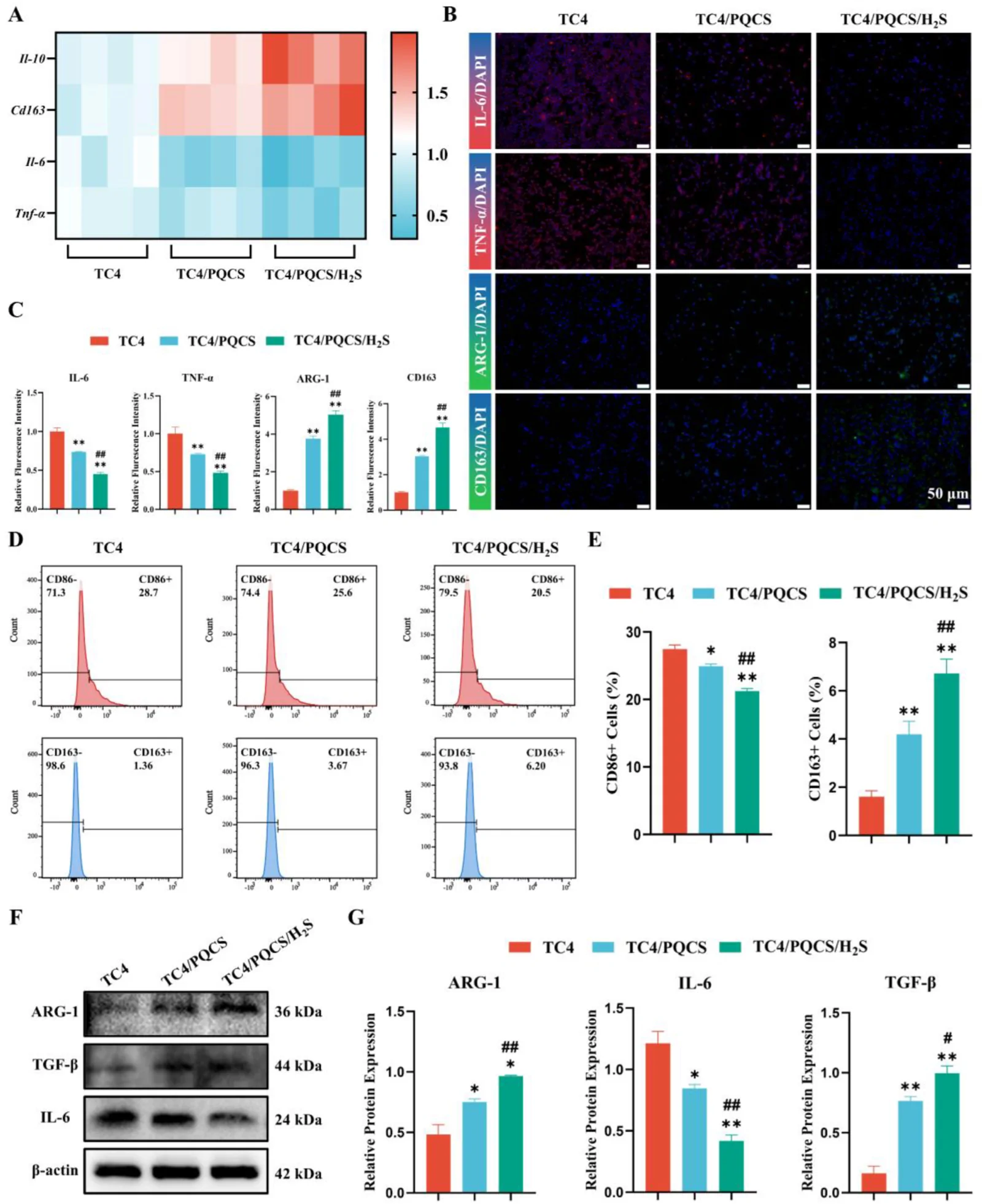
Immunomodulatory effects of the modified substrates on macrophage polarization. (A) Heatmap visualization of the relative mRNA expression of anti-inflammatory genes (*Il-10* and *Cd163*) and pro-inflammatory genes (*Il-6* and *Tnf-α*) in RAW264.7. (B-C) Immunofluorescence images and quantitative results of IL-6, TNF-α, ARG-1, and CD163 in RAW264.7. (D-E) Flow cytometry and quantitative analysis of the percentage of CD86^+^ (M1 phenotype) and CD163^+^ (M2 phenotype) cells. (F-G) Western blot and quantitative results of ARG-1, TGF-β, and IL-6 in RAW264.7. Data are presented as mean ± SD (n = 3). *p < 0.05 and **p < 0.01 versus TC4; ^#^*p* < 0.05 and ^##^*p* < 0.01 versus TC4/PQCS.

The modulation of macrophage polarization by the TC4/PQCS/H_2_S was evaluated via RT-qPCR analysis. The transcriptional levels of M1 markers (*Il-6* and *Tnf-α*) decreased progressively from the TC4 group to the TC4/PQCS group, reaching the lowest levels in the TC4/PQCS/H_2_S group (Fig. 7A). Conversely, the expression of M2 transcript markers (*Il-10* and *Cd163*) showed a stepwise increase, with the most upregulation observed in the TC4/PQCS/H_2_S group. Immunofluorescence staining corroborated these findings (Fig. 7B and C). Regarding the staining of M1 markers (IL-6, TNF-α), the fluorescence intensity was weakest in the TC4/PQCS/H_2_S group, followed by the TC4/PQCS group, both being markedly lower than in the TC4 group. In contrast, for M2 markers (ARG-1 and CD163), both the fluorescence intensity and the proportion of positive cells increased with the surface modification, with the strongest intensity detected in the TC4/PQCS/H_2_S group. Quantitative data obtained from flow cytometry further supported this immunomodulatory trend (Fig. 7D and E). The percentage of CD86^+^ M1 macrophages decreased sequentially in the order of the TC4, TC4/PQCS, and TC4/PQCS/H_2_S, while the proportion of CD163^+^ M2 cells exhibited the opposite pattern. Western blot analysis at the protein level was consistent with these observations (Fig. 7F and G). Compared with the TC4 and TC4/PQCS groups, the TC4/PQCS/H_2_S group showed significantly higher expression of anti-inflammatory proteins (ARG-1, TGF-β) alongside lower expression of the pro-inflammatory protein (IL-6). Collectively, these findings suggest that the TC4/PQCS/H_2_S coating promotes macrophage polarization from the M1 toward the M2 phenotype, indicating a favorable anti-inflammatory effect *in vitro*.

### 2.8 *In vivo* anti-infectious and immunomodulatory effects

**Fig. 8:**
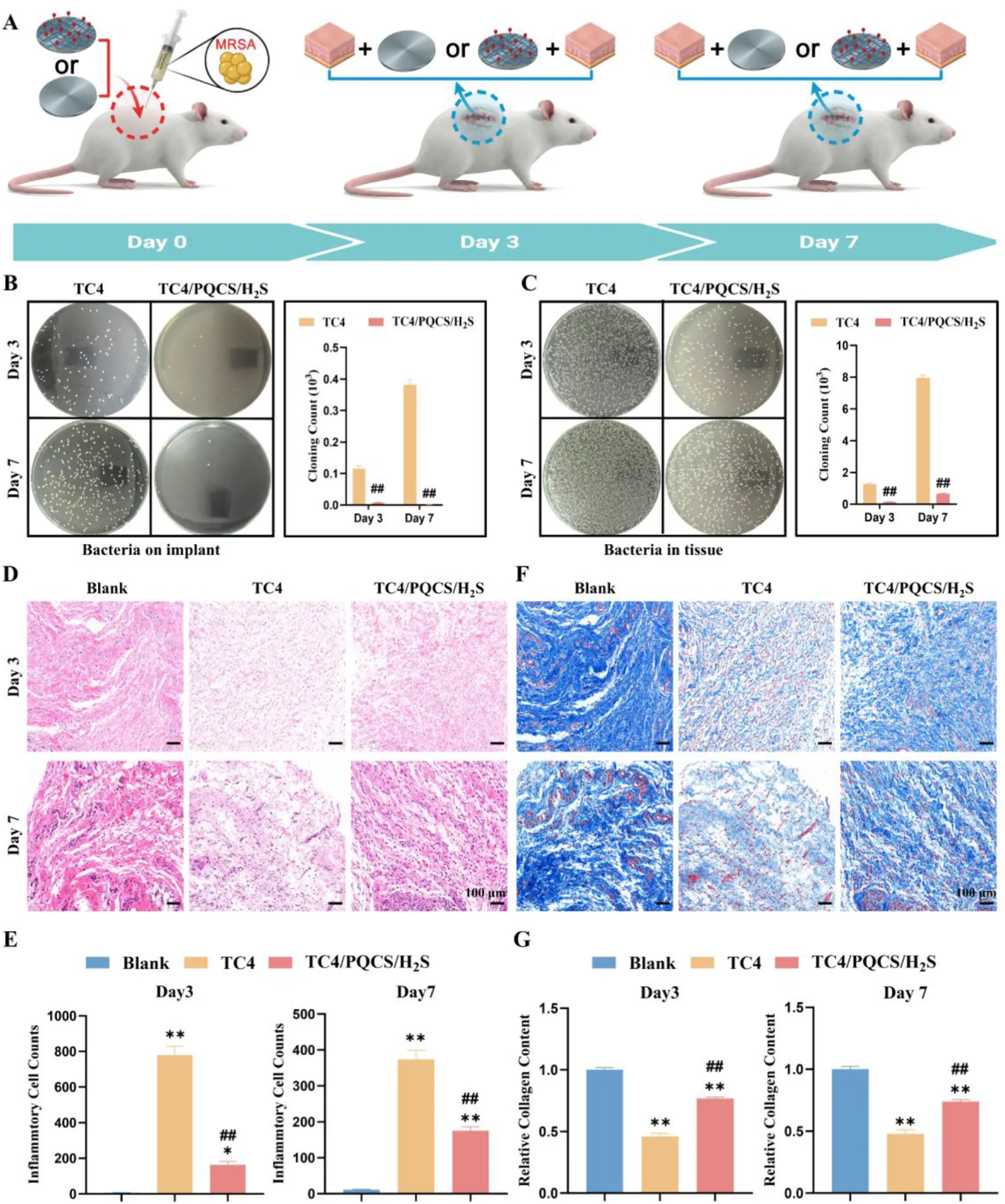
*In vivo* anti-infectious and anti-inflammatory activity. (A) Graphic illustration of the experimental protocol involving MRSA infection and subcutaneous implantation in rats. (B-C) Photographs of bacterial colonies and quantitative analysis of bacteria derived from the implants and surrounding tissues at day 3 and day 7. (D-E) H&E staining images and quantitative analyses of inflammatory cell counts of the skin tissues surrounding the implant. (F-G) Masson staining images and quantitative analysis of relative collagen content. Data are presented as mean ± SD (n = 5). *p < 0.05 and **p < 0.01 versus Blank; **^##^***p* < 0.01 versus TC4.

To further validate the therapeutic potential of the functionalized surface in infection control and microenvironment modulation, a MRSA-infected rat subcutaneous co-implantation model was employed (Fig. 8A).

Compared with the TC4 group, the TC4/PQCS/H_2_S group showed a significant decrease (**p < 0.01) in CFU counts on the implant surface and in the surrounding tissue (Fig. 8B and C). These results suggest that the TC4/PQCS/H_2_S coating not only reduces bacterial colonization on its surface but also actively clears pathogens disseminated into the surrounding tissue. Histological analysis further delineated the dynamic process of inflammatory resolution and tissue healing concurrent with infection control. On days 3 and 7, the blank group exhibited mild inflammatory cell infiltration, whereas both the TC4 and TC4/PQCS/H_2_S groups showed acute inflammatory cell infiltration (Fig. 8D and E). Notably, the TC4/PQCS/H_2_S group exhibited significantly reduced inflammatory cell infiltration compared with the TC4 group. Masson staining results further supported these observations (Fig. 8F and G). The blank group displayed tightly arranged and organized collagen fibers, whereas the TC4 group showed lighter collagen staining alongside fragmented and disrupted fiber architecture, which was attributed to early bacterial invasion and subsequent inflammatory infiltration. In contrast, the TC4/PQCS/H_2_S group exhibited favorable collagen deposition, characterized by a more densely packed and orderly fiber arrangement by day 7.

**Fig. 9:**
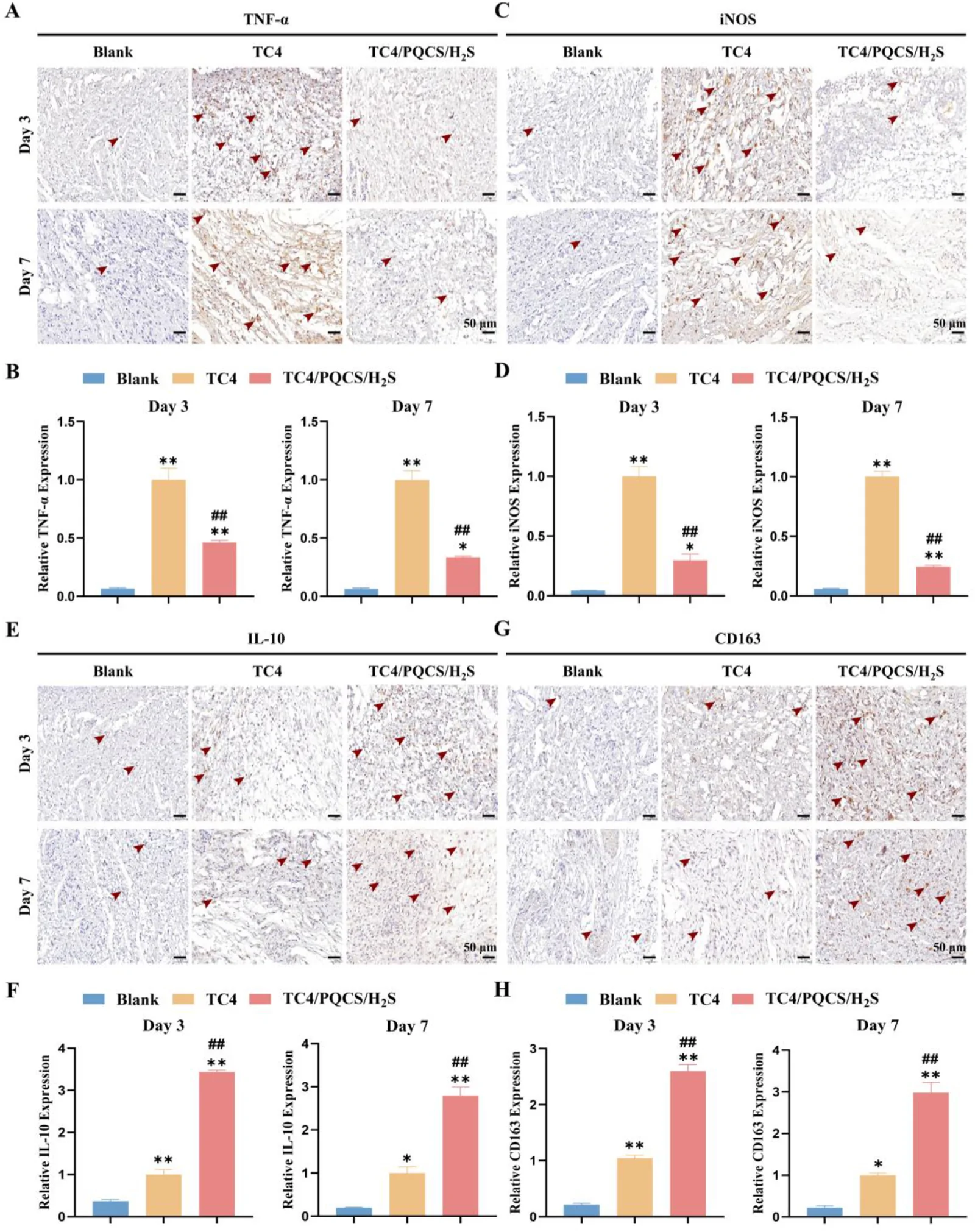
Immunohistochemical evaluation of the inflammatory response in the peri-implant tissues. Immunohistochemical staining images and corresponding quantitative analysis of (A-B) TNF-α, (C-D) iNOS, (E-F) IL-10, and (G-H) CD163 expression in the surrounding tissues at day 3 and day 7. Data are presented as mean ± SD (n = 5). *p < 0.05 and **p < 0.01 versus Blank; ^##^*p* < 0.01 versus TC4.

Immunohistochemical staining further corroborated these findings. On days 3 and 7, the TC4/PQCS/H_2_S group exhibited downregulation of pro-inflammatory M1 macrophage markers (TNF-α and iNOS) (Fig. 9A–D) and concurrent upregulation of M2 macrophage markers (IL-10 and CD163) (Fig. 9E–H) compared with the TC4 group. Collectively, these *in vivo* results demonstrate that the TC4/PQCS/H_2_S coating exerts antibacterial activity and immunomodulatory effects within infected microenvironments.

### 2.9 TC4/PQCS/H_2_S modulated macrophage polarization via PI3K-Akt pathway

Given the immunomodulatory effects and M2 polarization observed both *in vitro* and *in vivo*, RNA sequencing (RNA-seq) was performed to further elucidate how the TC4/PQCS/H_2_S coating modulates the local immune microenvironment and promotes macrophage phenotypic transition. This transcriptomic analysis aimed to identify the underlying signaling pathways involved in TC4/PQCS/H_2_S-mediated M2 macrophage polarization.

**Fig. 10:**
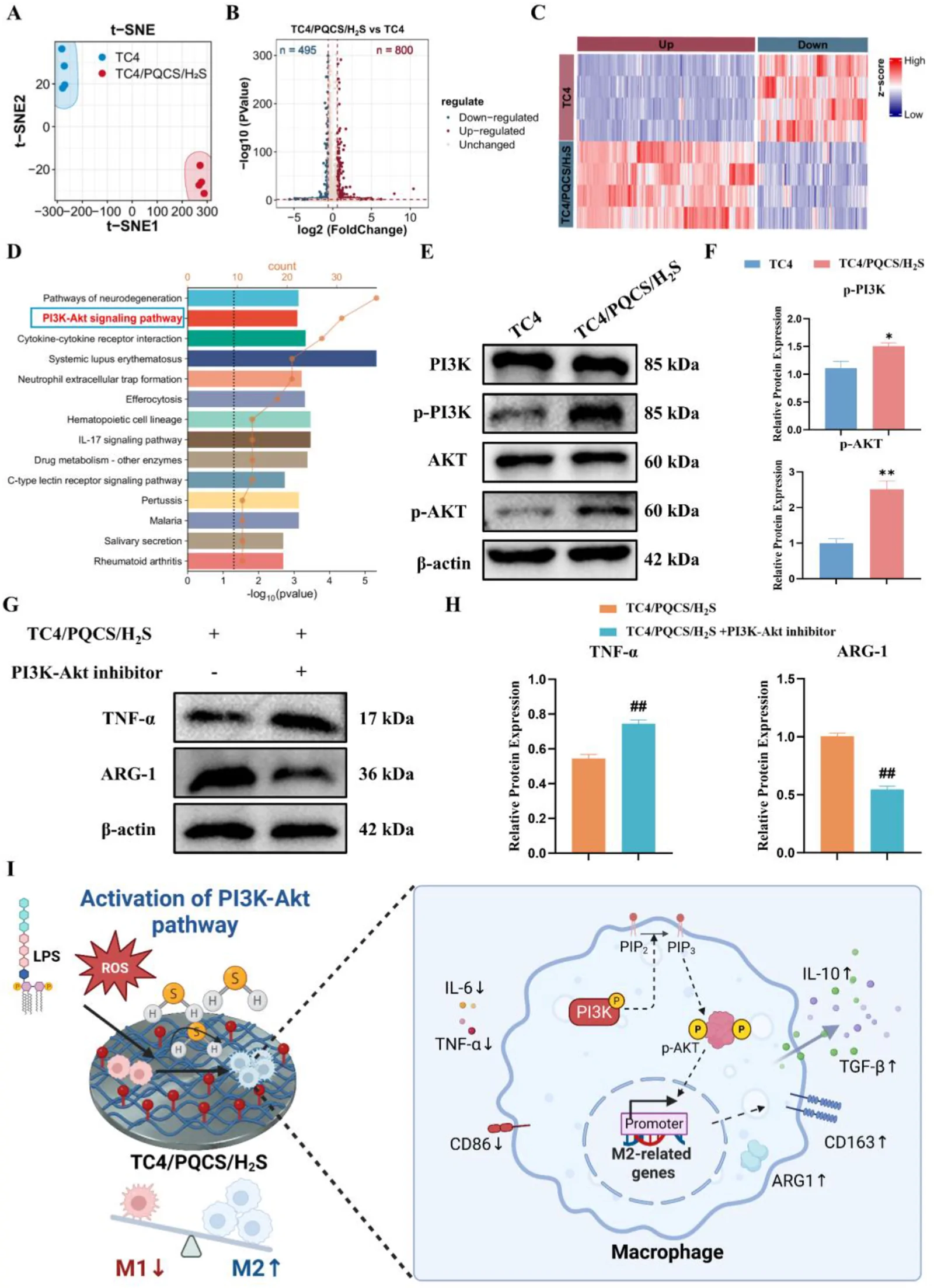
Elucidation of the immunomodulatory mechanism via the PI3K-Akt signaling pathway. (A) t-SNE plot showed distinct clustering between TC4 and TC4/PQCS/H_2_S groups. (B) Volcano plot of differentially expressed genes (DEGs) between TC4 and TC4/PQCS/H_2_S groups. (C) Heatmap of hierarchically clustered DEGs. (D) KEGG pathway enrichment analysis showed the significant enrichment of the PI3K-Akt signaling pathway. (E-F) Western blot and quantitative analysis of PI3K, p-PI3K, AKT, and p-AKT in RAW264.7 cells cultured on different samples. (G-H) Western blot and quantitative analysis of TNF-α and ARG-1 expression in RAW264.7 cultured on TC4/PQCS/H_2_S surfaces with (+) or without (−) a PI3K-Akt inhibitor (LY294002). (I) The underlying mechanism of the TC4/PQCS/H_2_S in PI3K-Akt pathways, which was created with BioRender.com. Data are presented as mean ± SD (RNA-seq: n = 4; Western blot: n = 3) *p < 0.05 and **p < 0.01 versus TC4; ^##^*p* < 0.01 versus TC4/PQCS/H_2_S.

Firstly, t-distributed stochastic neighbor embedding (t-SNE) revealed a distinct separation between the TC4 and TC4/PQCS/H_2_S groups (Fig. 10A), indicating that the introduction of the PQCS matrix and H_2_S donor induced a fundamental shift in the transcriptomic profiles of macrophages. This shift was further visualized by a volcano plot (Fig. 10B) identifying differentially expressed genes (DEGs), and a hierarchical clustering heatmap providing an intuitive overview of the expression patterns (Fig. 10C). To identify the signaling cascades regulated by the TC4/PQCS/H_2_S coating, Kyoto Encyclopedia of Genes and Genomes (KEGG) pathway enrichment analysis was conducted based on the identified DEGs. Of particular significance, the PI3K-Akt signaling pathway emerged as one of the enriched pathways (Fig. 10D). To validate these transcriptomic findings and assess the activation status of this pathway, the protein expression levels of key kinases were evaluated by Western blot (Fig. 10E and F). The ratios of p-PI3K to PI3K and p-AKT to AKT were markedly increased in the TC4/PQCS/H_2_S group compared with the TC4 group. To further investigate whether the PI3K-Akt signaling pathway is functionally involved in TC4/PQCS/H_2_S-induced macrophage polarization, rescue experiments were performed employing a specific PI3K-Akt inhibitor (LY294002). RT-qPCR analysis showed that LY294002 reversed the polarization phenotype, with increased expression of the M1 markers (*Tnf-α* and *Cd86*) and decreased expression of the M2 markers (*Arg-1* and *Cd163*) (Fig. S12A). This attenuation was further corroborated by flow cytometry, as evidenced by an increased percentage of CD86^+^ M1 macrophages and a decreased proportion of CD163^+^ M2 macrophages (Fig. S12B and C). Consistently, Western blot analysis further revealed a downregulated expression of ARG-1 and an upregulated expression of TNF-α following PI3K-Akt inhibition (Fig. 10G and H).

As a fundamental regulator of cellular survival and immunomodulation, the PI3K-Akt signaling pathway plays a pivotal role in moderating macrophage polarization [49]. When macrophages are stimulated by bioactive signals from the functionalized substrate, PI3K is phosphorylated and activated, generating lipid messengers that recruit AKT to the plasma membrane, where it is subsequently phosphorylated to p-AKT. Activated AKT then modulates downstream transcriptional activities to suppress pro-inflammatory cytokines, specifically IL-6, while upregulating anti-inflammatory and tissue-repair genes, such as TGF-β and ARG-1 [50]. The elevated levels of p-PI3K and p-AKT observed in the TC4/PQCS/H_2_S group are therefore consistent with the activation of this signaling pathway. Collectively, these results suggest that the TC4/PQCS/H_2_S coating drives macrophage polarization toward the M2 phenotype, a process closely linked to the activation of the PI3K-Akt signaling pathway (Fig. 10I).

Although the TC4/PQCS/H_2_S coating exhibits multiple advantageous properties, translating the coatings into clinical practice remains challenging. Achieving sustained therapeutic effects under the complex physiological conditions of the oral cavity requires further optimization of coating durability and prolonged ROS-responsive H_2_S release to combat peri-implant mucosal inflammation. Another challenge lies in more precisely decoupling the independent biological roles of the released H_2_S from the subtle variations in surface roughness, charge density, and molecular structure introduced by donor conjugation, which may collectively exert synergistic effects on the observed biological outcomes. Furthermore, while the rat subcutaneous implantation model used in this study provides valuable preliminary evidence for soft tissue response and immunomodulation, it does not fully recapitulate the complex oral environment, where implants are subjected to masticatory loading, salivary fluid dynamics, polymicrobial challenges. Therefore, establishing more clinically relevant animal models, such as the jawbone-implantation model, is essential for further verifying the soft tissue sealing ability and long-term immunomodulatory effects of the coating.

## 3 Conclusion

In this study, we developed a surface modification strategy for dental implants integrating quaternized chitosan with ROS-responsive H_2_S release to address the challenges associated with bacterial invasion and inflammation. By integrating quaternized chitosan with a stimuli-responsive H_2_S donor, the coating enables on-demand H_2_S release under ROS while maintaining stable interfacial properties. Rather than acting through a single pathway, this system provides a coordinated regulation of the peri-implant microenvironment, including enhanced cell attachment, effective antibacterial activity, and modulation of oxidative stress and immune responses. Overall, this responsive multifunctional coating holds great promise for improving soft‑tissue sealing and the long‑term success of dental implants.

## 4 Materials and methods

### 4.1 Materials

Chitosan (CS, molecular weight of 100 kDa, deacetylation degree of 85%) was provided by Jinan Haidebe Marine Biological Engineering Co., Ltd., China. Glycidyl trimethylammonium chloride (GTMAC), N-hydroxysuccinimide (NHS), 1-ethyl-(3-dimethylaminopropyl) carbodiimide (EDC), phloretic acid (PA), and sodium periodate were purchased from Shanghai Aladdin Biochemical Technology Co., Ltd., China. Tyrosinase was purchased from Beijing Solarbio Science and Technology Co., Ltd., China. P-Benzylboronic acid pinacol ester bromide was purchased from Shanghai Macklin Biochemical Co., Ltd., China. Thiourea, 2,2′-dipyridyl disulfide, and 3-thiopropionic acid were obtained from Shanghai Aladdin Biochemical Technology Co., Ltd., China. Commercial titanium alloy disks (Ti-6Al-4V, Φ10 × 1 mm, Φ16 × 1 mm) were supplied by Weihai Wego Jericom Biomaterials Co., Ltd., China. Sprague-Dawley rats (male, 8 weeks, 170-220 g) were purchased from Guangdong Weitonglihua Experimental Animal Technology Co., Ltd., China.

Animal experiments were performed in accordance with the Chinese Animal Welfare Guidelines and Experimental Protocols, approved by the Animal Ethics Committee of Guangdong Hua Wei Testing Co., Ltd. (NO: 202508002; HWT-BG-117b), and complied with the National Research Council's Guide for the Care and Use of Laboratory Animals. The sex of the animals did not affect the results of the experiment.

### 4.2 Synthesis and characterization of PQCS

Phloretic acid-modified quaternized chitosan (PQCS) was synthesized through a two-step procedure. First, chitosan (CS, 1 g) was dissolved in deionized water (36 mL) with acetic acid (180 μL) under stirring at 25 °C for 8 h. GTMAC solution (10 mL, 0.16 g/mL) was then added, and the reaction was continued at 55 °C for 24 h. After centrifugation at 10000 rpm for 5 min to remove insoluble material, the supernatant was dialyzed against deionized water for 72 h and freeze-dried to obtain quaternized chitosan (QCS). In the subsequent grafting step, QCS (0.2 g) was dissolved in a mixture of deionized water (20 mL) and N, N-dimethylformamide (10 mL). Separately, EDC (0.5 g), NHS (0.4 g), and PA (0.03 g) were dissolved in N, N-dimethylformamide (10 mL) and activated at 25 °C for 30 min. This activated solution was then added to the QCS solution. Following pH adjustment to 5.5, the reaction proceeded for 24 h. The final product was purified by dialysis against deionized water and concentrated using a 30 wt % polyethylene glycol solution to yield a 2 wt % PQCS solution. The chemical structure of PQCS was confirmed by proton nuclear magnetic resonance (^1^H NMR) spectroscopy (Bruker AVII-400 MHz, Switzerland) in D_2_O. The substitution degree of QCS was determined by using a titration method for Cl^−^. The chemical structure of PQCS was characterized using an ultraviolet-visible (UV-vis) spectrophotometer (Shimadzu, Japan).

### 4.3 Synthesis and characterization of H_2_S donor

3 g of p-benzylboronic acid pinacol ester bromide was dissolved in dry ethanol (30 mL) under N_2_. 1 g of thiourea was added to the solution. After reaction for 5 h, the mixture was concentrated via rotary evaporation to obtain the thiouronium intermediate, followed by dissolving in NaOH solution (0.1 g/mL, 20 mL). After refluxing for 1 h, the mixture was cooled and mixed with HCl to form a white precipitate. The aqueous layer was extracted, and the organic layer was separated and dried to obtain the crude product, which was then purified using silica gel chromatography to obtain compound 1. 1 g of compound 1 and 1.8 g of 2,2′-dipyridyl disulfide were dissolved in dry methanol (20 mL). After reaction for 12 h, the mixture was concentrated to obtain the crude product, followed by purification by silica gel chromatography to obtain the compound 2.1 g of compound 2 and 0.3 g of 3-thiopropionic acid were dissolved in dry methanol (10 mL). After reaction for 4 h, the mixture was concentrated, and the crude product was purified by silica gel chromatography to obtain the H_2_S donor. The chemical structure of the H_2_S donor was characterized by ^1^H NMR spectroscopy using CDCl_3_ as the solvent.

### 4.4 Fabrication of TC4/PQCS/H_2_S

Tyrosinase was dissolved in 0.5 mL of the phosphate buffer solution (PBS, pH = 7.4) and added to a 2 wt % PQCS solution at a final concentration of 1 kU/mL. Then, 1 mL of solution was added to a tube to immerse each TC4. After reacting for 24 h at 37 °C, the PQCS decorated TC4 (TC4/PQCS) was removed, rinsed with PBS, and dried in air. The dried TC4/PQCS was immersed in the activated solution containing the H_2_S donor (0.2 g), EDC (0.6 g), and NHS (0.4 g). After 48 h, the TC4 was fully rinsed and dried to yield the TC4/PQCS/H_2_S.

### 4.5 Characterization

The surface chemical composition of the samples was investigated using X-ray photoelectron spectroscopy (XPS, Thermo Fisher Scientific K-Alpha, USA) with an Al K-Alpha X-ray source. Crystalline structure and phase composition were analyzed by X-ray diffraction (XRD, Bruker D8 Advance, Germany) operated in symmetric reflection mode, with a scanning range of 4-40° at a rate of 1° per min. The chemical structure was characterized using an attenuated total reflectance-Fourier transform infrared (ATR-FTIR) spectrometer (Bruker, Germany). Surface morphology and elemental distribution were examined using scanning electron microscopy (SEM, ZEISS Sigma300, Germany) coupled with energy-dispersive X-ray spectroscopy (EDS, Apollo, EDAX, USA). The surface roughness was quantitatively assessed by atomic force microscopy (AFM, Multimode 8, Bruker, Germany).

The wettability of the samples was evaluated through water contact angle measurements using a contact angle goniometer (CAMKSV021733, Nunc, Finland). Protein adsorption behavior was assessed by incubating samples in a 2 mg/mL bovine serum albumin (BSA) solution at 37 °C for 4 h, followed by rinsing with PBS. The adsorbed protein was subsequently desorbed using a 10% sodium dodecyl sulfate (SDS) solution and quantified by measuring the absorbance at 562 nm with a UV-vis spectrophotometer.

To evaluate the mechanical stability and durability of the coatings under simulated oral environments, the TC4/PQCS and TC4/PQCS/H_2_S samples were subjected to adhesion and stability tests. For the ultrasonication test, the samples were immersed in ultrapure water and subjected to three cycles of ultrasonic cleaning using a high-frequency digital ultrasonic cleaner (KQ-200 TDE, Kunshan, China). For the peeling test, a 3M Scotch adhesive tape (adhesion strength > 1.23 N/cm) was applied to the sample surface with uniform pressure and then peeled off in the same direction three times. After these treatments, the surface morphology, crystalline structure of chitosan, and surface wettability were characterized by SEM, XRD, and water contact angle measurements, respectively.

To further assess the stability and structural integrity of the coatings under clinically relevant conditions, the TC4/PQCS and TC4/PQCS/H_2_S samples were immersed in artificial saliva (R41109, Yuanye, Shanghai) and PBS solutions with different pH values (pH 7.4 and pH 5.5) culture medium at 37 °C for 7 and 14 days. After the designated immersion periods, the surface morphology at both 7 and 14 days was characterized by SEM. XPS analysis was performed on samples after 14 days of immersion to investigate the surface chemical composition. XRD was employed to evaluate the crystalline structure of chitosan on the coating surface after 14 days of immersion. Additionally, the surface wettability of samples at both 7 and 14 days was evaluated by water contact angle measurements.

### 4.6 Cell culture

The mouse leukemic monocyte macrophage cell line RAW264.7 and human gingival fibroblasts (HGFs) were obtained from American Type Culture Collection (Manassas, VA, USA). Human gingival epithelial cells (HGECs) were obtained from Xiamen Immocell Biotechnology Co., Ltd. (Xiamen, China). HGFs and HGECs were cultured in α-Minimum Essential Medium (α-MEM), while RAW264.7 macrophages were cultured in Dulbecco's Modified Eagle Medium (DMEM). Both media were supplemented with 10% fetal bovine serum (FBS) and 1% penicillin-streptomycin (Gibco). All cells were maintained in a humidified incubator at 37 °C with 5% CO_2_. Cells were seeded on test substrates in 12-/24-well plates at optimal densities, with medium replenished every 2-3 days.

### 4.7 *In vitro* biocompatibility evaluation

#### 4.7.1 Cell proliferation assay

The proliferative activity of HGFs, HGECs, and RAW264.7 macrophages on different material surfaces was quantitatively assessed using a Cell Counting Kit-8 (CCK-8; Dojindo, Japan) assay. The HGFs, HGECs, and RAW264.7 macrophages were cultured with various groups of specimens for 1, 3, and 5 days. After incubation periods, the culture medium was replaced with a fresh medium containing 10% (v/v) CCK-8 reagent and incubated for an additional 2 h. Following incubation, 100 μL of the supernatant from each well was transferred to a new 96-well plate. The absorbance of the resulting solution was measured at a wavelength of 450 nm using a microplate reader to determine the formazan dye concentration, which is proportional to the number of viable cells.

#### 4.7.2 Live/dead staining assay

Cell viability and membrane integrity were qualitatively analyzed using a live/dead cell staining kit (Beyotime Biotechnology, China). HGFs, HGECs, and RAW264.7 macrophages were seeded on the test materials at a density of 3.0 × 10^4^ cells/well and cultured for 3 days. After the incubation period, cells were stained according to the manufacturer's instructions using a solution containing calcein-AM and propidium iodide (PI). Viable cells, characterized by esterase activity and intact membranes, convert the non-fluorescent calcein-AM to intracellular green fluorescence. In contrast, non-viable cells with compromised membranes are permeable to PI, which binds to nucleic acids and emits red fluorescence. The stained cells were immediately visualized using a fluorescence microscope.

#### 4.7.3 Cytoskeletal and nuclear staining

To investigate cell morphology, adhesion, and cytoskeletal architecture, HGFs, HGECs, and RAW264.7 macrophages were cultured on material surfaces (3.5 × 10^4^ cells/well) for 24 h. After that, cells were fixed with 4% paraformaldehyde (PFA) for 30 min and permeabilized with 0.5% Triton X-100 for 10 min. Then, cells were incubated with TRITC-conjugated phalloidin (1: 200, Yeasen Biotechnology, China) for 90 min in the dark, followed by counterstaining with 4′,6-diamidino-2-phenylindole (DAPI; 5 μg/mL, Solarbio, China) for 5 min. The stained samples were stored in PBS and imaged using a fluorescence microscope.

#### 4.7.4 Hemolysis test

The hemocompatibility of the materials was evaluated via a hemolysis test. Fresh rat blood was anticoagulated with sodium citrate (blood-to-anticoagulant ratio of 9:1, v/v) and subsequently diluted with normal saline at a 4:5 (v/v) ratio. The test materials, with a defined surface-area-to-volume ratio of 1.25 cm^2^/mL, were immersed in the diluted blood and incubated at 37 °C for 1 h. Normal saline and distilled water served as the negative and positive controls, respectively. Post-incubation, the mixtures were centrifuged at 1500 rpm for 10 min. The absorbance of the supernatants was measured at 545 nm using a UV-Vis spectrophotometer. The hemolysis ratio was calculated using the following formula:

Hemolysisratio(%)=(A1−A2)(A3−A2)×100%

A_1_, A_2_, and A_3_ represent the absorbance values of the samples, negative control, and positive control, respectively.

### 4.8 The adhesion assay of HGECs and HGFs

#### 4.8.1 RT-qPCR for adhesion-related genes

HGECs (1 × 10^5^ cells per well) and HGFs (1 × 10^5^ cells per well) were cultured on the prepared substrates for 24 h. Total RNA was extracted and reverse-transcribed into cDNA using commercial kits (Z-press RNA Purification Kit and 5 × Evo M-MLV RT Master Mix, respectively; EZBioscience, China). Subsequently, RT-PCR was performed using 2 × Color SYBR Green Master Mix (EZBioscience, China) under the following cycling conditions: 95 °C for 5 min; 40 cycles of 95 °C for 10 s and 60 °C for 30 s. Relative gene expression levels, normalized to β-actin, were calculated using the 2^–ΔΔCt^ method. The corresponding primer sequences are provided in Table S1.

#### 4.8.2 Immunofluorescence staining for the marker of adhesion

HGECs (5 × 10^4^ cells per well) and HGFs (5 × 10^4^ cells per well) were cultured on the prepared substrates for 24 h. After incubation, cells cultured on coverslips were fixed with 4% paraformaldehyde for 15 min, permeabilized with 0.1% Triton X-100, and blocked with a solution containing BSA. The cells were then incubated with primary antibodies overnight at 4 °C, followed by incubation with appropriate fluorophore-conjugated secondary antibodies for 1 h at room temperature in the dark. For HGECs, primary antibodies against LN332 and ITGB4 were incubated, while for HGFs, primary antibodies against VCL and ITGB1 were incubated. Finally, the nuclei were counterstained with DAPI, and the stained samples were mounted for imaging under a fluorescence microscope. The corresponding antibodies are provided in Table S2.

#### 4.8.3 Transwell migration assay

Cell migration was evaluated using Transwell chambers with 8.0 μm pore size membranes (Corning, USA). HGECs and HGFs were first cultured on material samples for 24 h. The cells were then harvested and resuspended in serum-free medium. A total of 200 μL of cell suspension (3.5 × 10^4^ cells) was added to the upper chamber. The lower chamber was filled with 600 μL of complete medium containing 20% FBS. After 48 h of incubation, the inserts were retrieved, and the apical medium was aspirated. Non-migrated cells on the upper surface were carefully removed using a cotton swab. The inserts were subsequently rinsed twice with PBS, fixed in 4% paraformaldehyde for 15 min, and washed three additional times with PBS. The fixed cells were then stained with 0.1% crystal violet for 15 min, rinsed with tap water to terminate the staining, and left to air-dry. Migrated cells were visualized and photographed under an optical microscope.

### 4.9 *In vivo* biosafety evaluation and cell adhesion assay

A subcutaneous implantation model was established to evaluate the cell adhesion and soft tissue compatibility at the implant–tissue interface. Animals were divided into three experimental groups: TC4, TC4/PQCS, and TC4/PQCS/H_2_S. Zoletil 50 (1 mg/100 g) and Xylazine (1.2 mg/100 g) were administered via intramuscular injection to induce anesthesia, after which all surgical procedures were carried out under sterile conditions. A 1.0 cm longitudinal incision was created on the dorsum, and a subcutaneous pocket was prepared by blunt dissection to accommodate the sterilized cylindrical specimens (Φ10 × 1 mm). At 7 and 14 days post-implantation, the animals were euthanized, and the implants with the surrounding soft tissues attached were harvested. The implant–tissue interface tissues were then collected for subsequent histological and immunohistochemical evaluation. For biosafety assessment, major organs (heart, liver, spleen, lung, and kidney) were harvested at 14 days post-implantation for hematoxylin and eosin (H&E) staining.

To evaluate the soft tissue compatibility and cytoskeletal organization at the implant–tissue interface, H&E staining and Masson staining were performed on the implant–tissue interface sections to assess the morphological features and collagen deposition. Paraffin-embedded tissues were sectioned at a thickness of 4 μm, followed by deparaffinization, rehydration, and staining according to standard protocols.

For immunofluorescence staining, the sections underwent deparaffinization and rehydration, followed by antigen retrieval. Then, they were incubated with primary antibodies against VCL and ITGB1 at 4 °C overnight, and subsequently with appropriate fluorophore-conjugated secondary antibodies (Proteintech, Wuhan, China). Nuclei were counterstained with DAPI, and the stained sections were mounted and imaged under a fluorescence microscope. For immunohistochemical staining, the sections were incubated with primary antibodies against COL1A1 at 4 °C overnight, followed by incubation with horseradish peroxidase-conjugated secondary antibodies (Proteintech, Wuhan, China). Immunoreactive signals were visualized using diaminobenzidine (DAB) substrate, and nuclei were counterstained with hematoxylin. The stained sections were then mounted and imaged under a light microscope. Detailed information of antibodies used is provided in Table S2.

### 4.10 Evaluation of antioxidant capacity

#### 4.10.1 Intracellular ROS detection

RAW264.7 macrophages, HGFs, and HGECs at a density of 3 × 10^4^ cells/mL were seeded on the surfaces of samples and incubated for 24 h. After replacing the serum-free medium with H_2_O_2_ (250 μM for HGFs and HGECs, and 100 μM for RAW264.7), cells were cultured for 8 h. They were then stained with DCFH-DA (Beyotime Biotechnology, China) at 37 °C for 30 min in the dark, and fluorescence images were captured using a fluorescence microscope.

#### 4.10.2 RT-qPCR for Nrf2 pathway markers

RAW264.7 macrophages were seeded on the sample surfaces at a density of 1.0 × 10^5^ cells/mL and cultured for 24 h. The medium was then replaced with serum-free medium containing H_2_O_2_ (100 μM), and the cells were cultured for an additional 24 h. The RT-qPCR experiment, described in section 4.8.1, was conducted to evaluate the expression levels of Nrf2 pathway-related genes in RAW264.7 cells. The primer sequences for these genes are listed in Table S1.

#### 4.10.3 Immunofluorescence staining for Nrf2 pathway markers

RAW264.7 macrophages (4 × 10^4^ cells/mL) were seeded on different samples and cultured for 24 h. The medium was then replaced with serum-free H_2_O_2_-containing medium (100 μM), and the cells were cultured for an additional 24 h. Immunofluorescence staining was then performed as described in Section 4.8.2 to detect the expression of KEAP1, NRF2, and HO-1. The antibodies used are listed in Table S2.

#### 4.10.4 Nrf2 inhibition assay

An Nrf2 inhibition assay was performed to verify the involvement of the Nrf2 pathway. RAW264.7 macrophages were pre-incubated with ML385 (2 μM) for 2 h, followed by stimulation with H_2_O_2_ (100 μM) for 8 h. Intracellular ROS levels were detected following the procedure described in Section 4.10.1.

### 4.11 Measurement of H_2_S from TC4/PQCS/H_2_S

#### 4.11.1 H_2_S ROS-responsive release

The TC4/PQCS/H_2_S was immersed in 1 mL of phosphate buffer saline (PBS, pH = 7.4) with or without H_2_O_2_ (300 μM). After a pre-determined time interval, 50 μL of supernatant was transferred to a 96-well plate, followed by the addition of Zn(COO)_2_ solution (10 μL, 1 wt%), FeCl_3_ solution in 1.2 M HCl (20 μL, 30 mM), and N, N-dimethyl-p-phenylenediamine in 7.2 M HCl (20 μL, 20 mM). The mixture was incubated for 20 min, and its optical density at 670 nm was measured using a microplate reader.

#### 4.11.2 Intracellular H_2_S release

HGFs, HGECs, and RAW264.7 were seeded directly onto the sterilized TC4 and TC4/PQCS/H_2_S pre-placed in a 12-well plate at a density of 1 × 10^5^ cells per well and cultured for 12 h to allow for cell attachment. Subsequently, the culture medium was replaced with a fresh medium containing lipopolysaccharide (LPS, 5 μg/mL for HGFs and HGECs, and 1 μg/mL for RAW264.7) to stimulate the cells for 12 h. Following stimulation, the cells were washed with PBS and then incubated with a working solution containing the H_2_S-sensitive fluorescent probe WSP-5 (50 μM) and the nuclear stain Hoechst 33342 (10 μg/mL) for 30 min at 37 °C in the dark. After incubation, the cells were gently rinsed with PBS to remove excess dye, and intracellular H_2_S release was visualized and imaged using an inverted fluorescence microscope.

### 4.12 *In vitro* antimicrobial activity evaluation

The antibacterial efficacy of the material was evaluated against both aerobic and anaerobic bacterial strains. The samples were exposed to suspensions of Gram-positive Methicillin-resistant *Staphylococcus aureus* (MRSA, ATCC43300) and Gram-negative *Escherichia coli* (*E. coli*, ATCC25922) (concentration of 1 × 10^6^ CFU/mL) and incubated under standard aerobic conditions for 24 h. Subsequent bacterial colonization on the material surfaces was qualitatively analyzed using SEM and live/dead staining. Furthermore, quantitative assessment of antibacterial activity was performed by enumerating viable bacteria via the standard plate count method following the incubation period. The antimicrobial activity against anaerobic bacteria, including *Porphyromonas gingivalis* (*P. gingivalis*, ATCC33277) and *Fusobacterium nucleatum* (*F. nucleatum*, ATCC25586), was also quantitatively determined using the plate count method. Briefly, samples were incubated with bacterial suspensions (1 × 10^7^ CFU/mL) at 37 °C for 24 h under anaerobic conditions. After incubation, the adherent bacteria were detached from the samples by gentle ultrasonication and vortex agitation in sterile PBS. The harvested bacterial suspensions were serially diluted, plated onto appropriate agar plates, and cultivated anaerobically. The resulting colony-forming units were counted to evaluate the bacterial survival rate on each sample. Colony-forming units were then counted according to the National Standard of China GB/T 4789.2 protocol, and the antibacterial rates were calculated using the formula:

Antibacterialrates(%)=(A‐B)/A×100%

A and B represent the average number of viable bacteria on the TC4 and the test groups (TC4/PQCS and TC4/PQCS/H_2_S), respectively. The detailed experimental methods were described in our previous articles [51,52].

### 4.13 Crystal violet staining for *in vitro* biofilm biomass quantification

MRSA, *E. coli*, *P. gingivalis*, and *F. nucleatum* were separately cultured to the logarithmic growth phase. The bacterial suspensions were then adjusted to a concentration of 1 × 10^8^ CFU/ml using appropriate media. Sterile material samples were placed into 12-well plates for biofilm formation. Each well received 1 ml of the corresponding bacterial suspension (1 × 10^8^ CFU/ml), ensuring that the samples were fully submerged. For MRSA and *E. coli*, the plates were incubated at 37 °C under aerobic conditions for 3 days. For *P. gingivalis* and *F. nucleatum*, the plates were incubated at 37 °C under anaerobic conditions for 5 days. The culture media were refreshed every 24 h to maintain nutrient supply and eliminate planktonic bacteria.

After biofilm formation, the titanium samples were gently rinsed three times with sterile PBS to wash off non-adherent bacteria. For quantification of biofilm biomass, the samples were fixed with methanol for 15 min and then air-dried. Staining was performed using 0.1% (w/v) crystal violet solution at room temperature for 20 min. The samples were subsequently washed three times with PBS to remove unbound dye and air-dried for 1 h. The crystal violet retained on the samples was dissolved in 1 ml of anhydrous ethanol per well. The absorbance of the resulting solution was measured at 590 nm using a microplate reader. Biofilm biomass was presented as relative biomass in comparison to the control group. The biofilm eradication rate was determined using the following formula:

Biofilmeradicationrate(%)=(1‐ODtestODcontrol)×100%

### 4.14 Evaluation of *in vivo* antimicrobial efficacy and infection resistance

*In vivo* antimicrobial efficacy and infection resistance were assessed using a subcutaneous infection model. The animals were randomly divided into three groups: (1) Blank Control (sham operation without implantation and without bacterial injection), (2) TC4 group, and (3) TC4/PQCS/H_2_S group. All surgeries were performed under aseptic conditions after the animals were anesthetized by intramuscular injection of Zoletil 50 (1 mg/100 g) and Xylazine (1.2 mg/100 g). A dorsal incision (1.0 cm) was made, and a subcutaneous pocket was created via blunt dissection. Sterilized cylindrical samples (Φ10 × 1 mm) were implanted into the pockets in the TC4 and TC4/PQCS/H_2_S groups. After the incision was sutured, 50 μL of MRSA suspension (1 × 10^6^ CFU/mL) was injected into the implantation site in these two groups. Animals were euthanized at predetermined time points (3 days and 7 days), and implants with surrounding tissues were harvested for subsequent analysis.

To directly quantify the *in vivo* antibacterial efficacy, complementing the preceding *in vitro* assays, the bacterial loads both on the explanted samples and within the surrounding peri-implant tissues were assessed. The explanted samples were gently rinsed with PBS and subsequently subjected to ultrasonication in a known volume of sterile PBS to detach the adherent bacteria. Concurrently, the harvested peri-implant tissues were homogenized in PBS. The resulting bacterial suspensions from the implants and the tissue homogenates were then serially diluted, plated on agar, and incubated for colony counting.

### 4.15 *In vivo* anti-inflammatory and immunomodulatory evaluation

To evaluate the tissue response at the implant–tissue interface, H&E staining and Masson staining were performed on tissue sections. H&E staining was used to assess inflammatory cell infiltration, while Masson staining was employed to evaluate collagen deposition. In addition, immunohistochemical staining was performed to analyze macrophage polarization markers (TNF-α, iNOS, IL-10, and CD163), following the procedure described in Section 4.9. Antibodies are listed in Table S2.

### 4.16 *In vitro* modulation of macrophage immune response

#### 4.16.1 Analysis of M1/M2 macrophage polarization gene expression in RAW264.7 macrophages by RT-qPCR

RAW264.7 macrophages (2.5 × 10^5^ cells per well) were first cultured on different samples for 48 h. Subsequently, the cells were stimulated with 1 μg/mL LPS for an additional 24 h, reaching a total culture duration of 72 h. An RT-qPCR study was conducted as described in sections 4.8.1. The corresponding primer sequences are provided in Table S1.

#### 4.16.2 Immunofluorescence staining for the macrophage polarization-relative marker

RAW264.7 macrophages (3.5 × 10^4^ cells per well) were first cultured on different samples for 48 h. Subsequently, the cells were stimulated with 1 μg/mL LPS for an additional 24 h, reaching a total culture duration of 72 h. Immunofluorescence staining was then performed to detect the expression of IL-6, TNF-α, ARG-1, and CD163, following the procedure described in Section 4.8.2. Antibodies are listed in Table S2.

#### 4.16.3 Flow cytometric analysis of M1/M2 macrophage markers in RAW264.7 macrophages

RAW264.7 macrophages (3 × 10^5^ cells per well) were cultured on different samples for 48 h, followed by a 24-h stimulation with 1 μg/mL LPS. After treatment, cells were harvested, washed with cold PBS, and blocked with an Fc receptor blocking reagent (eBioscience, China). The cells were then stained with fluorochrome-conjugated antibodies against specific M1 and M2 surface markers or corresponding isotype controls for 30 min on ice in the dark. Following staining, cells were washed, resuspended in cold flow cytometry staining buffer, and analyzed immediately on a BD FACSCantoII Analyzer (BD Biosciences). Data analysis was performed using FlowJo software (V.10.4). Detailed antibody information is provided in Table S2.

#### 4.16.4 Western blotting analysis for the macrophage polarization-associated proteins

After an initial incubation of 48 h on different samples, RAW264.7 macrophages (seeded at 2.5 × 10^5^ cells per well) were exposed to 1 μg/mL LPS for an additional 24 h. Total protein was extracted from treated RAW264.7 macrophages using RIPA lysis buffer (Beyotime, China) supplemented with protease and phosphatase inhibitors. The lysates were centrifuged, and the protein concentration in the supernatant was determined with a BCA assay kit (Beyotime, China). Equal amounts of protein (15 μg per lane) were separated by SDS-PAGE on 10% or 12.5% gels and subsequently transferred to PVDF membranes. After blocking with 5% non-fat milk or BSA in TBST for 1 h at room temperature, the membranes were incubated overnight at 4 °C with specific primary antibodies against IL-6, TGF-β, ARG-1, and β-actin. Following this, the membranes were probed with an HRP-conjugated secondary antibody (Proteintech, China) for 1 h at room temperature. Protein bands were finally visualized using an enhanced chemiluminescence (ECL) substrate and a chemiluminescence imaging system. Detailed antibody information is provided in Table S2.

### 4.17 Analysis of macrophage polarization signaling pathways

#### 4.17.1 RNA sequencing and analysis of macrophage polarization signaling pathways

RAW 264.7 (seeded at 2.5 × 10^5^ cells per well) macrophages were cultured on TC4 and TC4/PQCS/H_2_S substrates for 48 h, followed by stimulation with 1 μg/mL LPS for an additional 24 h. Total RNA was extracted using TRIzol reagent, and its integrity was confirmed (RNA Integrity Number ≥ 7.0) using an Agilent 2100 Bioanalyzer. Sequencing libraries were constructed from qualified RNA samples and sequenced on an Illumina NovaSeq 6000 platform to generate 150-bp paired-end reads. The raw reads were processed to remove low-quality sequences and adapters. Clean reads were then aligned to the mouse reference genome (GRCm39) using HISAT2. Differential gene expression analysis between groups was performed with the DESeq2 R package. Genes meeting the thresholds of an adjusted p-value (FDR) < 0.05 and |log2 (fold change)| ≥ 1 were identified as statistically significantly differentially expressed genes (DEGs). t-distributed stochastic neighbor embedding (t-SNE) was performed to visualize the overall transcriptional differences between samples. Kyoto Encyclopedia of Genes and Genomes (KEGG) enrichment of DEGs was implemented in the clusterProfiler R package (V.4.7.1.2) [53]. Enriched pathways with adjusted p-value below 0.05 were considered as significantly enriched by DEGs.

#### 4.17.2 PI3K-Akt signaling pathway analysis and inhibition assay

Western blotting for macrophage polarization signaling pathways was conducted as described in Section 4.16.4. Antibodies are provided in Table S2. To further confirm the role of the PI3K-Akt pathway in macrophage polarization, rescue experiments were conducted using a PI3K-Akt inhibitor (LY294002, 50 μM). After treatment, the expression of downstream polarization markers was evaluated at both the mRNA and protein levels. RT-qPCR was performed to detect the expression of *Cd86*, *Tnf-α*, *Cd163*, and *Arg-1*, following the procedure described in Section 4.8.1. Primer sequences are provided in Table S1. Flow cytometry was conducted to analyze the surface expression of CD86 and CD163, as described in Section 4.16.3. Western blotting was performed to detect the expression of TNF-α and ARG-1, following the procedure described in Section 4.16.4. Detailed antibody information is provided in Table S2.

### 4.18 Statistical analysis

Statistical analyses were conducted using GraphPad Prism 9 software (GraphPad Software, La Jolla, CA, USA). All quantitative data are expressed as mean ± standard deviation (SD). Prior to analysis, the distribution assumptions of the data were evaluated. Data normality was assessed using the Shapiro-Wilk test, and variance homogeneity was examined via the Brown-Forsythe test. For comparisons between two groups, the two-tailed Student's t-test was performed. For multi-group comparisons, one-way or two-way analysis of variance (ANOVA) was applied as appropriate. Tukey's post hoc test was subsequently employed to handle multiple comparisons and adjust *p*-values. In all statistical tests, a *p*-value less than 0.05 was considered statistically significant.

## Data availability

Data will be made available on request.

## CRediT authorship contribution statement

**Yu Xie:** Data curation, Investigation, Visualization, Writing – original draft. **Dandan Xia:** Conceptualization, Funding acquisition, Investigation, Project administration, Supervision, Writing – review & editing. **Zhen Chen:** Data curation, Formal analysis, Methodology, Validation, Writing – review & editing. **Haitong Wu:** Software, Validation. **Lepu Zeng:** Methodology, Software. **Ting Zhong:** Validation. **Wenxue Chen:** Validation. **Xinchen Du:** Conceptualization, Data curation, Funding acquisition, Investigation. **Ming Ma:** Conceptualization, Data curation, Investigation, Project administration, Supervision, Validation, Visualization, Writing – review & editing. **Xuechao Yang:** Conceptualization, Project administration, Resources, Supervision.

## Ethics approval and consent to participate

Animal experiments were performed in accordance with the Chinese Animal Welfare Guidelines and Experimental Protocols, approved by the Animal Ethics Committee of Guangdong Hua Wei Testing Co., Ltd. (NO: 202508002; HWT-BG-117b), and complied with the National Research Council's Guide for the Care and Use of Laboratory Animals. The sex of the animals did not affect the results of the experiment.

## Declaration of competing interest

The authors declare the following financial interests/personal relationships which may be considered as potential competing interests: Given her role as editorial board member for Bioactive Materials, Dandan Xia was not involved in the peer review of this article and had no access to information regarding its peer review. Full responsibility for the editorial process for this article was delegated to another journal editor. All authors declare that there are no competing interests.
