# Supplementary material for: ROS-responsive H2S-releasing quaternized chitosan dental implant coating for improved soft tissue integration, antibacterial and immunomodulatory capacities
Source: Bioact Mater. 2026 Aug 20;67:597–619. doi: 10.1016/j.bioactmat.2026.07.056 (PMC13522379; doi:10.1016/j.bioactmat.2026.07.056)
Supplement: Supplementary file 1 [file mmc1.docx]

**Supplementary Information**

**ROS-responsive H_2_S-releasing quaternized chitosan dental implant coating for improved soft tissue integration, antibacterial and immunomodulatory capacities**

**Fig. S1.** (A) Synthetic route of PQCS. (B) ^1^H NMR spectrum of PQCS. (C) UV-vis spectra of deionized water, PA, QCS, and PQCS solutions.

**Fig. S2.** (A) Synthetic route of H_2_S donor. (B) ^1^H NMR spectrum of H_2_S donor.

**Fig. S3.** (A) EDS elemental mapping images of TC4 and TC4/PQCS. (B) XRD pattern of TC4, TC4/PQCS, and TC4/PQCS/H_2_S.

**Fig. S4.** SEM images showing the surface morphology of the TC4/PQCS and TC4/PQCS/H_2_S coatings after different simulated treatments: (A) high-frequency ultrasonication, (B) 3M tape peeling tests, and prolonged immersion in artificial saliva, PBS (pH 7.4), or PBS (pH 5.5) for (C) 7 days and (D) 14 days.

**Fig. S5.** (A) XPS survey spectra of the TC4/PQCS/H_2_S coating after 14 days of immersion in artificial saliva, PBS (pH 7.4), or PBS (pH 5.5). (B–F) XRD patterns of TC4/PQCS and TC4/PQCS/H_2_S coatings following (B) ultrasonication, (C) 3M tape peeling, or 14-day immersion in (D) artificial saliva, (E) PBS (pH 7.4), and (F) PBS (pH 5.5). (G–N) Water contact angle measurements of the coatings immediately after (G) ultrasonication and (H) 3M tape peeling, as well as after immersion in various media for (I–K) 7 days and (L–N) 14 days. Data are presented as mean ± SD (n = 3). ^***^*p* < 0.001 versus TC4.

**Fig. S6. *In vivo* biocompatibility evaluation.** Representative H&E staining images of rat’s heart, liver, spleen, kidney, and lung to evaluate biosafety and inflammation-responsive functionality.

**Fig. S7. Assessment of ITGB1 expression after long-term fluid immersion.** (A) Representative immunofluorescence images of ITGB1 expressions in HGFs in each group after 14 days of immersion in (A) artificial saliva, (B) PBS (pH 7.4), or (C) PBS (pH 5.5). (D) Corresponding quantitative analysis of the relative fluorescence intensity of ITGB1 under various immersion conditions. Data are presented as mean ± SD (n = 3). ^*^*p* < 0.05 and ^**^*p* < 0.01 versus TC4.

**Fig. S8. *In vivo* immunohistochemical staining of the material-tissue interface.** (A) Immunohistochemical staining images of COL1A1 expression at the material-tissue interface at day 7 and day 14. (B) Quantitative analysis of the relative COL1A1 expression level at the material-tissue interface. Data are presented as mean ± SD (n = 5). ^**^*p* < 0.01 versus TC4.

**Fig. S9. Antibacterial evaluation of different groups after treatment under various conditions.** Representative photographs of bacterial colonies and corresponding antibacterial rates after (A, B) ultrasonication, (C, D) 3M tape peeling, and 14‑day immersion in (E, F) artificial saliva, (G, H) PBS (pH 7.4), or (I, J) PBS (pH 5.5). Data are presented as mean ± SD (n = 3). ^**^*p* < 0.01 versus TC4.

**Fig. S10.** ***In vitro* antimicrobial and antibiofilm activities.** (A, B) CFUs photographs and antibacterial ratio of *P. gingivalis* and *F. nucleatum* after co-incubation with different samples. (C-D) Crystal violet staining images and corresponding absorbance statistics of MRSA*, E. coli, P. gingivalis, and F. nucleatum* biofilms after co-incubation with different samples. Data are presented as mean ± SD (n = 3). **^**^***p* < 0.01 versus TC4.

**Fig. S11.** (A) Representative immunofluorescence images of KEAP1 expression in RAW264.7 cells. (B) Relative mRNA expression levels of *Keap1* in RAW264.7 cells. (C–E) Quantitative analysis of the relative fluorescence intensity of (C) KEAP1, (D) nuclear NRF2, and (E) HO-1 in RAW264.7 cells. (F, G) Representative immunofluorescence images and corresponding quantitative analysis of intracellular ROS levels in RAW264.7 cells cultured on TC4/PQCS/H_2_S samples with or without the Nrf2 inhibitor (ML385) after H_2_O_2_ treatment. Data are presented as mean ± SD (n = 3). ^**^*p* < 0.01.

**Fig. S12.** (A) Relative mRNA expression levels of pro-inflammatory genes (*Cd86* and *Tnf-α*) and anti-inflammatory genes (*Cd163* and *Arg-1*) in RAW264.7 cultured on TC4/PQCS/H_2_S surfaces with (+) or without (-) a PI3K-Akt inhibitor (LY294002). (B-C) Flow cytometry and quantitative analysis of the percentage of CD86^+^ (M1 phenotype) and CD163^+^ (M2 phenotype) cells in RAW264.7 cultured on TC4/PQCS/H_2_S surfaces with (+) or without (-) a PI3K-Akt inhibitor (LY294002). Data are presented as mean ± SD (n = 3). ^*^*p* < 0.05 and ^**^*p* < 0.01.

**Table. S1 Primer sequences for quantitative PCR (qPCR) analysis. This table lists the target genes, including their forward and reverse primer sequences.**

| **Gene** | **Forward Primer** | **Reverse Primer** |
| --- | --- | --- |
| ***COL17A1*** | TCAACCAGAGGACGGAGTCA | TCGACTCCCCTTGAGCAAAC |
| ***LN332*** | GAGACGCCATAAACAACGGC | CTCGGAAAGCAGGCGTAGAA |
| ***ITGB4*** | TGTCCATCCCCATCATCCCT | CCCGATGGAGAGCGTAGAAC |
| ***ITGA6*** | TGCTGTTGGTTCCCTCTCAG | TGTTTTCTGGCGGAGGTCAA |
| ***VCL*** | CTGAACCAGGCCAAAGGTT | GATCTGTCTGATGGCCTGCT |
| ***FAK*** | CGACAGCAACAGGAAATGG | TGCCTCGAGAGAGTCTCACAT |
| ***ITGB1*** | GCCTGTTTACAAGGAGCTGAA | CTGACAATTTGCCGTTTTCC |
| ***ACTA2*** | TAAGACGGGAATCCTGTGAAGC | TGTCCCATTCCCACCATCAC |
| ***TGFB1*** | TGTCACCGGAGTTGTGCGGC | GGCCGGTAGTGAACCCGTTG |
| ***EDN1*** | ACGGCGGGGAGAAACCCACT | ACGGAACAACGTGCTCGGGA |
| ***COL1A1*** | TCTAGACATGTTCAGCTTTGTGGAC | TCTGTACGCAGGTGATTGGTG |
| ***FN*** | CGGAGAGACAGGAGGAAATAGCCCT | TTGCTGCTTGCGGGGCTGTC |
| ***VEGFA*** | GAGCCTTGCCTTGCTGCTCTAC | CACCAGGGTCTCGATTGGATG |
| ***Keap1*** | GACCTGGACTTTCGTAGCCC | CCGGGTCATAGCATTCCACA |
| ***Nrf2*** | AGAGTGATGGTTGCCCACTT | GCGTGCTCAGAAACCTCCTT |
| ***Gpx4*** | GCCTCGCAATGAGGCAAAAC | CAAACTGGTTGCAGGGGAAG |
| ***Gpx1*** | AAGGCTCACCCGCTCTTTAC | GCACACCGGAGACCAAATGA |
| ***Sod1*** | TTCTCGTCTTGCTCTCTCTGG | CTTCTGCTCGAAGTGGATGGT |
| ***Sod2*** | GAGAGCAGCGGTCGTGTAAA | AGCCTCGTGGTACTTCTCCT |
| ***Hmox-1*** | GAACCCAGTCTATGCCCCAC | GGCGTGCAAGGGATGATTTC |
| ***Il-10*** | CTTACTGACTGGCATGAGGATCA | GCAGCTCTAGGAGCATGTGG |
| ***Cd163*** | ATGCTTCCATCCAGTGCCTC | CACAAACCAAGAGTGCCGTG |
| ***Arg-1*** | AGCTCTGGGAATCTGCATGG | ATGTACACGATGTCTTTGGCAGATA |
| ***Il-6*** | CTGCAAGAGACTTCCATCCAG | AGTGGTATAGACAGGTCTGTTGG |
| ***Tnf-α*** | CGGGCAGGTCTACTTTGGAG | ACCCTGAGCCATAATCCCCT |
| ***Cd86*** | ATGAGAAAGCAGCAAAGCAAG | TTACTGGTGGCACAGGGTCTG |
| ***β-actin (M)*** | GTGACGTTGACATCCGTAAAGA | GCCGGACTCATCGTACTCC |
| ***β-actin (H)*** | CACCATTGGCAATGAGCGGTTC | AGGTCTTTGCGGATGTCCACGT |

**Table. S2 Antibodies for western blotting and other related experiments**

| **Antibody** | **Source** | **Cat No.** |
| --- | --- | --- |
| LN332 | Immunoway, USA | YT2525 |
| ITGB4 | Immunoway, USA | YM9258 |
| COL1A1 | Abcam, USA | ab138492 |
| VCL | Proteintech, China | 66305-1-Ig |
| ITGB1 | Proteintech, China | 12594-1-AP |
| KEAP1 | Immunoway, USA | YM8283 |
| NRF2 | Immunoway, USA | YM8624 |
| HO-1 | Immunoway, USA | YM8337 |
| TNF-α | Abcam, USA | ab183218 |
| iNOS | Proteintech, China | 18985-1-AP |
| IL-10 | Abcam, USA | ab189392 |
| CD163 | Proteintech, China | 16646-1-AP |
| IL-6 | Abcam, USA | ab290735 |
| ARG-1 | Proteintech, China | 16001-1-AP |
| TGF-β | Proteintech, China | 21898-1-AP |
| CD163-PE | Biolegend, USA | 155307 |
| CD86-FITC | Biolegend, USA | 105005 |
| Isotype Control-FITC | Biolegend, USA | 400505 |
| Isotype Control-PE | Biolegend, USA | 400507 |
| PI3K | Cell Signaling Technology, USA | #4292 |
| pPI3K | Cell Signaling Technology, USA | #4228 |
| AKT | Cell Signaling Technology, USA | #4691 |
| pAKT | Cell Signaling Technology, USA | #4060 |
| β-actin | Proteintech, China | 20536-1-AP |
